# Sanitation and Collective Efficacy in Rural Cambodia: The Value Added of Qualitative Formative Work for the Contextualization of Measurement Tools

**DOI:** 10.3390/ijerph17010001

**Published:** 2019-12-18

**Authors:** Allison P. Salinger, Gloria D. Sclar, James Dumpert, Davin Bun, Thomas Clasen, Maryann G. Delea

**Affiliations:** 1 Hubert Department of Global Health, Rollins School of Public Health, Emory University, Atlanta, GA 30322, USA; 2 Learning & Documentation, WaterAid Cambodia, Phnom Penh 12207, Cambodia; jwdumpert@gmail.com (J.D.); bundavin7@gmail.com (D.B.); 3 Department of Environmental Health, Rollins School of Public Health, Emory University, Atlanta, GA 30322, USA; gloria.sclar@emory.edu (G.D.S.); thomas.f.clasen@emory.edu (T.C.); mdelea@emory.edu (M.G.D.)

**Keywords:** collective efficacy, water, sanitation, and hygiene (WASH), behavior change, collective action, community-based interventions, participatory development approaches, factor analysis, social context

## Abstract

Community-level action may be required to achieve the levels of sanitation uptake necessary for health gains. Evidence suggests that collective action is influenced by collective efficacy (CE)—a group’s belief in its abilities to organize and execute action to achieve common goals. The extent to which it is necessary to fully contextualize existing CE measurement tools, in order to conduct meaningful assessments of the factors influencing CE perceptions, is not well understood. This study examines the value added of contextualizing an existing CE measurement tool using qualitative formative research. We employed a modified grounded theory approach to develop a contextualized CE framework based on qualitative data from rural Cambodian villages. The resulting framework included sub-constructs that were pertinent for the rural Cambodian context for which an existing, hypothesized framework did not account: perceived risks/benefits, action knowledge, shared needs/benefits, and external accountability. Complex confirmatory factor analyses indicated that contextualized models fit the data better than hypothesized models for women and men. This study demonstrates that inductive, qualitative research allows community-derived factors to enhance existing tools for context-specific CE measurement. Additional research is needed to determine which CE factors transcend contexts and could, thus, form the foundation of a general CE measurement tool.

## 1. Introduction

According to the most recent data from the Joint Monitoring Program (JMP) for Water Supply and Sanitation, 41% of the Cambodian population of approximately 16 million practice open defecation, a far larger proportion than in any neighboring southeast Asian nation [1]. Thailand, for example, eliminated open defecation in 2015 [1].

In rural Cambodia, where 77% of the population reside, lack of access to improved sanitation facilities poses a major challenge to reducing open defecation [2]. Only 31% of the rural population has access to improved sanitation facilities, compared to 88% of the urban population in Cambodia [3]. The links between unsafe sanitation, poor hygiene practices, and both morbidity and mortality are well supported, albeit with a significant degree of heterogeneity, in the published literature [4]. In Cambodia, 238 disability-adjusted life years (DALYs) per 100,000 were attributable to unsafe sanitation during 2017 compared to 10 DALYs per 100,000 in Thailand in the same year [5].

Initiated in 2011, the Cambodia Rural Sanitation and Hygiene Improvement Program (CRSHIP) seeks to increase access to and use of improved latrines with handwashing facilities among rural target communities. The program’s implementing partners utilize participatory development approaches that focus on changing behavior and generating demand for latrines [6]. The first phase of the program (CRSHIP1) was implemented from 2011–2016, and reached 2027 villages; however, only 756 of these communities were declared open defecation free (ODF) by the Ministry of Rural Development [7]. Retrospective evaluations of CRSHIP1 identified social context as an important moderating factor for sanitation uptake [7].

### 1.1. Community-Level Sanitation

Evidence suggests that certain levels of water, sanitation, and hygiene (WASH) coverage and utilization are likely required to realize health gains through herd protection [8,9,10]. For instance, community-level sanitation usage rates of 60%–80% and 80% or more are associated with lower prevalence odds of active trachoma compared to communities that had sanitation usage rates of less than 20%. Thus, individuals and households may benefit from the sanitary investments and hygienic behaviors of others in their community [11].

Some argue that assessments of exposure–disease relationships at the individual level may be less meaningful than those conducted at the community level [4]. Achieving biologically consequential gains in sanitation status may, therefore, require community-centric or collective processes, which are influenced by interpersonal behavioral factors. Conceptualizing sanitation as a public good provides a rationale for examining how and why social context may moderate the effectiveness of community-based, participatory sanitation programs.

### 1.2. Collective Efficacy

Collective efficacy (CE) is a group’s shared belief in its abilities to organize and execute joint action required to achieve common goals [12]. Positive CE perceptions regarding the group’s ability and autonomy to achieve communal goals will improve a group’s motivation to pursue those goals. This enhanced motivation may, in turn, increase the group’s resilience in the face of obstacles and, thus, the likelihood of goal attainment [13,14]. Assessments that incorporate CE measurements may facilitate a more comprehensive understanding of the antecedents of collective action, particularly within the context of community-level sanitation programming. Assessments of social capital, alone, for example, would not fully consider the influence of multiple motivational factors on collective sanitation behaviors [15,16].

Tools for measuring CE have been developed for a variety of topics and populations [13,17,18]; few, however, have been deployed in rural developing contexts [19]. Measurement tools adapted from Bandura’s efficacy scale [20] ask respondents about their level of certainty (0%–100%) that their group can achieve various levels of performance regarding specific tasks [21]. Other tools identify domains of CE pertinent to the specific population, topic, or both and use Likert-type response scales to assess respondents’ agreement with statements that pertain to one or more of the identified domains [22].

### 1.3. Study Objectives

There is not yet sufficient evidence to determine whether any of the existing CE measurement tools can be used to make meaningful CE assessments in contexts outside of those in which the tools were developed. The purpose of this study was to examine the value added of contextualizing an existing CE measurement tool using qualitative formative research. Qualitative research can generate community-derived inputs that can then be incorporated into existing tools to conduct meaningful assessments of the factors influencing CE perceptions.

## 2. Methods

### 2.1. Study Design

**Figure 1:**
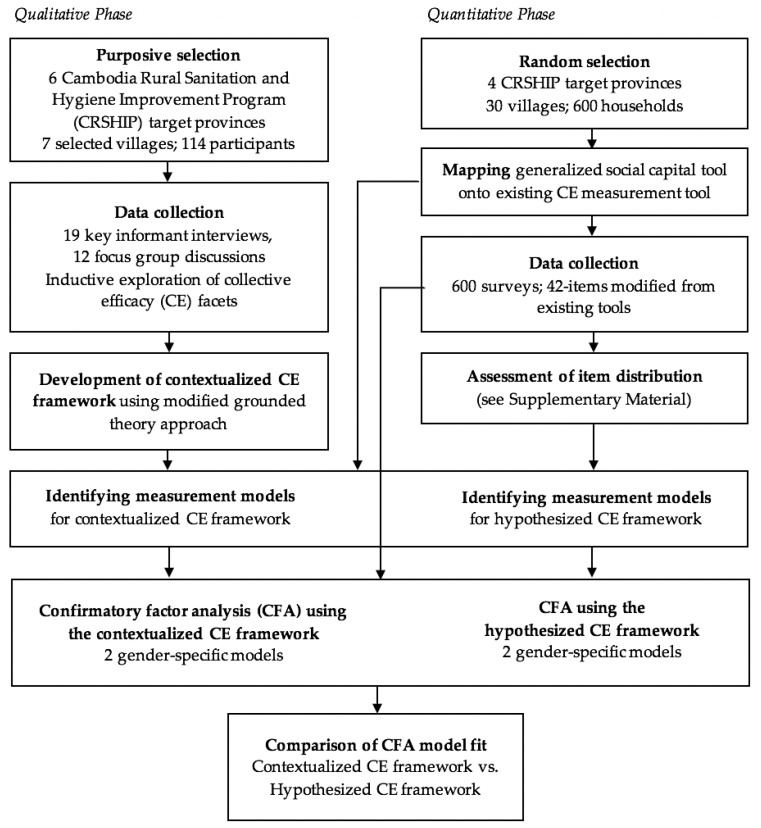
Concurrent triangulation mixed methods study design.

This study followed a concurrent triangulation mixed methods design to examine the value added of additional qualitative work to fully contextualize a CE measurement tool. This work was executed in two phases—a formative qualitative research phase and a subsequent quantitative research phase (Figure 1). We conducted qualitative formative research in CRSHIP communities to elicit perceptions representative of the rural Cambodian context and used resulting data to develop a contextualized CE framework. We then compared this contextualized CE framework to an existing, hypothesized CE framework that was based on theory and empirical evidence, and refined during two studies conducted in India and Ethiopia [23]. The hypothesized framework was locally adapted to account for differences in language and setting, but not further contextualized to reflect unique or differently nuanced factors influencing CE in the Cambodian context. Confirmatory factor analyses (CFAs) were conducted to evaluate the fitness of the two frameworks to survey data collected in CRSHIP target villages [24]. Evidence suggests that perceptions of CE and group performance may differ by gender, and that men and women may contribute differently to collective action situations [25,26]. Therefore, we conducted a gendered examination of CE and related factors (i.e., sub-constructs) by obtaining data from both men and women, and fitting gender-specific models. Our study methods were reviewed and approved by the National Ethics Committee for Health Research in Cambodia (NECHR reference 158), as well as reviewed and exempted by the Institutional Review Board of Emory University.

### 2.2. Qualitative Research Phase

#### 2.2.1. Sample Selection and Participant Recruitment

Six CRSHIP provinces (Kampong Speu, Kampong Thom, Kampot, Kandal, Kratie, and Takeo) were purposively selected for this study given their variation in socio-cultural factors (e.g., presence of minority ethnic groups, practice of minority religions), which allowed for a more encompassing examination of CE. CRSHIP-implementing partners selected one village in each province and connected the research team to local authorities. The research team conducted key informant interviews (KIIs) with the selected local authorities who then identified one active village member to serve as an additional key informant. Local authorities also helped recruit men and women from the village to participate in focus group discussions (FGDs). Participants were at least 18 years of age and residents of the CRSHIP target village; no inclusion criteria concerning latrine ownership or individual CRSHIP program participation were used. A total of 19 KIIs and 12 FGDs were conducted across seven villages in the six selected provinces; KIIs and FGDs were conducted in three rounds of data collection, with two provinces per round.

#### 2.2.2. Qualitative Data Collection

In each of the study villages, KIIs were conducted with one commune-level authority (commune chief or commune councilor), one village-level authority (village chief or sub-village chief), and one active community member. In Cambodia, a commune is one administrative unit above village. As such, commune-level authorities are uniquely positioned to provide perspective on both local events (i.e., endogenous influences) and national political and economic trends (i.e., exogenous influences) that may influence the uptake of community-based sanitation program initiatives. Village-level authorities were interviewed for their knowledge of CRSHIP program activities and the specific social context of the village. The active community member provided another perspective on village activities and context in an effort to reach saturation with the KII findings. Key informants were asked to share reflections on CRSHIP programming, perceptions of CE, and beliefs about external influences on the community. See File S2 in Supplementary Materials for the full KII question guide.

Two FGDs were conducted in each study village, one with men and one with women, in order to capture normative perceptions and shared experiences, as well as variation in community members’ opinions. Gender-segregated FGDs allowed for a comparison of findings derived predominantly from men to those derived predominantly from women. Participants were asked to share perspectives on community, perceptions of CE (see Table S2 in Supplementary Material for further details regarding the thematic areas we examined), and norms related to latrine ownership and use. The focus groups ranged in size from five to 10 participants with the exception of one FGD which included only three participants due to difficulty with participant recruitment during rice-planting season. See File S1 in Supplementary Materials for the full FGD question guide.

Oral consent to participate and be audio-recorded was obtained from all participants at the start of KIIs and FGDs. Interviews and FGDs were conducted in Khmer by a trained research assistant. An additional field officer provided real-time English translation of key points to allow the investigator an opportunity for follow-up on salient topics that arose during the course of the interview or discussion. Interviews typically lasted 60 to 90 min and were conducted in the commune office or the participant’s home. FGDs typically lasted 90 to 120 min and were conducted in a community member’s home or a central location such as the village pagoda or school. Between each round of data collection, changes were made to the KII and FGD guides to improve wording and translation, as well as to incorporate emergent concepts. The trained research assistant transcribed and translated interview recordings into English for analysis by the investigator.

#### 2.2.3. Qualitative Analysis

The qualitative phase followed a modified grounded theory approach to develop a CE framework. Grounded theory was selected as the analytical approach because it was important that the contextualized CE framework incorporated emergent concepts and was reflective of the rural Cambodian context [27]. However, the approach is considered a modified grounded theory approach because a combination of inductive and deductive codes was used [28,29].

Open coding was conducted by two investigators after each of the three rounds of data collection. Codes were then organized, compared, and formalized; the resulting codebook was applied by one analyst across all transcripts in a process of focused coding [30]. Transcripts were grouped according to gender to identify differences between the perceptions of women and men [27,29]. Codes were then grouped into categories and subcategories according to the dimensions and facets of CE to which they referred, respectively. The resulting conceptual framework was then verified using the concept-indicator model. This model verifies that each level of the framework is based on empirical indicators from the level below such that domains were grounded in dimensions, which were grounded in facets, grounded in codes, grounded in textual data [31].

### 2.3. Quantitative Research Phase

#### 2.3.1. Sampling Strategy

Four provinces were selected for inclusion in the quantitative research phase (Kampong Cham, Kampong Speu, Kandal, and Takeo) from among those targeted under CRSHIP; these provinces were selected for their proximity to the capitol to facilitate data collection. Within each of the four provinces, seven to eight villages were randomly selected such that the study sample included a total of 30 randomly selected villages. A total of 600 households were surveyed across the 30 selected villages (140–160 households from each of the four provinces of interest).

#### 2.3.2. Household Survey

WaterAid commissioned a household survey based on the World Bank’s Integrated Questionnaire for the Measurement of Social Capital [32]. We appended an additional 30 items to this survey to investigate other sub-constructs related to CE. These additional items were adapted from an existing CE survey consisting of 50 items [23]. We conducted a mapping exercise to determine which of the 50 items overlapped with the World Bank’s social capital questionnaire. Twenty such items were identified, dropped from the 50-item survey, and replaced with 12 items from the social capital questionnaire. The resulting CE measurement tool had 42 items (Table S2). Enumerators conducted the household surveys in Khmer. Verbal consent was obtained from all household survey respondents prior to survey administration.

#### 2.3.3. Confirmatory Factor Analysis

Confirmatory factor analysis is a latent variable modeling method. CFA assesses hypothesized relationships between observed variables and their underlying latent constructs by evaluating item–factor relationships and model fit indices [24]. We conducted complex CFA in Mplus7 software (Muthén and Muthén, Los Angeles, CA, USA) to compare four CE models: models with data from women and men respondents informed by the contextualized CE framework (Models 1 and 2, respectively) and by the hypothesized CE framework (Models 3 and 4, respectively). We then identified measurement models by determining which of the 42 items tapped to the CE dimensions in each framework.

Given all items had ordinal, categorical response options, a robust weighted least squares with mean and variance adjustment (WLSMV) estimation method based on polychoric correlation matrices was used to perform the CFA. Non-independence of observations within 30 village clusters was addressed through the use of a sandwich estimator [33]. In order to conduct the gender-specific analyses, the 600-household dataset was split by gender of the respondent (*N*_WOMEN_ = 410, *N*_MEN_ = 186).

The pattern of item–factor relationships (i.e., factor loadings) was examined for all factors in each of the four models. Although some sources caution against the use of thresholds, common guidelines can be used to facilitate interpretation of factor loadings (e.g., factor loadings >0.71 are considered excellent, >0.63 very good, >0.55 good, >0.45 fair, and >0.32 adequate) [34]. For the purposes of this study, we omitted any item with a factor loading with an absolute value <0.30 [24,34].

To compare model fit, we used root-mean-square error of approximation (RMSEA), the comparative fit index (CFI), and the Tucker–Lewis index (TLI), as well as the Kline method for assessing model fit using χ^2^ [35,36,37,38,39]. According to the Kline method, a good absolute model fit is indicated by a non-significant χ^2^ or a ratio of χ^2^ to degrees of freedom (df) that is less than 3:1 [39]. For RMSEA, values of 0.05 or less indicate good absolute model fit, and values between 0.05 and 0.08 indicate adequate absolute model fit [38]. For relative/incremental fit statistics (CFI and TLI), larger values indicate better model fit. Values of 0.90 or above indicate adequate relative model fit, while values of 0.95 and above indicate good relative model fit [35,36,37].

## 3. Results

### 3.1. Participant Demographics

A total of 114 individuals participated in the qualitative research activities. Almost half of them were women (46%), most owning a latrine (75%) and having a primary school education or lower (71%); about one-third (30%) were classified as poor or very poor by Cambodia’s Ministry of Planning (Table 1). In addition, 600 individuals responded to the household survey; four respondents were excluded from the analytical sample because their gender was not documented. Among the 596 included respondents, the majority were women (69%), owned a latrine (53%), and had a primary school education or lower (63%); about one-fourth (26%) were classified as poor or very poor by Cambodia’s Ministry of Planning (Table 1).

### 3.2. Contextualized CE Framework

The modified grounded theory approach yielded a contextualized, tiered framework for CE (Table 2). Four domains (social control, social cohesion, social capital, and motivational investment) were identified as the main latent variables influencing CE perceptions. Below, we outline examples from the qualitative findings for each domain identified.

#### 3.2.1. Social Control

Qualitative data provided rich evidence that social control was a component of CE in CRSHIP villages. Participants reported intervening to correct behaviors around which the community had strong normative beliefs. Conversely, participants described a “mind your own business” mentality when it came to sanitation-related behaviors, which had weaker normative beliefs. It was, however, acceptable for those community members who held a position of power in the social context of the village (e.g., older generations, local leaders) to intervene in some situations when it came to sanitation.

“Since I am a village member, it is hard to give them advice […] some people might say the latrines belong to them, so I do not need to advise them. They might talk back to me, so it is hard.”

(FGD with men, Kampong Speu province)

“If they haven’t built [a latrine] yet, I told them that they must; otherwise, I won’t sign when they need to make a loan. They have to promise me, and they follow it. I have to threaten them.”

(Village chief, age 65, man, Kandal province)

#### 3.2.2. Social Cohesion

Social cohesion arose in the qualitative findings through participants’ expressions of belonging and attachment to their community, as well as descriptions of social equity. Many participants reported equitable distribution of resources in their communities, with external aid preferentially distributed to the poorer households in the village.

Interviewer (I): “What if anything happens such as disaster including flooding or drought, does everyone in this village get the same assistance? […]”

Participant 2 (P2): “Village chief and commune chief can help when drought or flooding happen.”

I: “Does everyone get helped?”

P2: “Yes! They help everyone.”

I: “What about other people? Do you think everyone gets the same assistance?”

P3: “Yes! Everyone gets the same.”

I: “Why? Why does everyone get the same assistance?”

P *: “It’s because everyone faces the drought the same, that’s why we get the same assistance although it’s not much.”

* Participant indistinguishable

(FGD with women, Takeo province)

Participants also expressed that community members contribute resources to community development projects equitably, with a sliding scale of contribution allowed for those that were unable to afford the requested donation.

I: “How do people feel about contribution [to community development projects]?”

P: “Those people that don’t have money contribute half of the truck [of cement]. People have solidarity. Anyone that is rich, they contribute two trucks. We build the road in front of my home.”

I: “How do people feel when they contribute differently?”

P: “It’s not a matter. They have good communication! I say it’s good. They aren’t jealous. People contribute based on their ability.”

(Key informant, age 56, woman, Kampong Thom province)

While participants did not describe many instances of discrimination based on a community member’s socioeconomic status (SES), some participants did cite differences in SES as a barrier to collective action because families with high SES had different needs than families with low SES.

“People that have better SES do not want to join with people that have low SES. They are rich, so they have no interest in working together. It is easier to mobilize people that are in the same SES.”

(Village chief, age 70, man, Takeo province)

#### 3.2.3. Social Capital

Participants from all of the communities engaged in the qualitative research activities expressed social capital as a factor that influences their CE perceptions. Specifically, participants described strongly held norms of reciprocity in favor of contributing money and labor for the development of village infrastructure (e.g., roads, canals, schools), for the celebration of village ceremonies (e.g., weddings, funerals, religious ceremonies), and for providing assistance to neighbors in urgent need (e.g., times of sickness, flood, or fire).

However, the idea of contributing to the purchase or construction of another family’s household latrine was unacceptable to study participants, in large part because sanitation was not viewed as a public good. Participants specifically cited the following reasons as to why this type of collective action was unacceptable for sanitation: (1) personal property does not benefit the whole community; (2) neighbors are only able to provide aid to the poor for urgent matters; (3) the cost of a latrine or of providing latrines to all households in the village without a latrine was prohibitive; (4) non-governmental organizations (NGOs) would provide latrines so money is better spent on other community development projects; and (5) households without improved latrines likely do not care about sanitation nor want improved latrines.

I: “Why can people help each other in anything, but not latrine?”

P3: “It’s because it matters to the individual.”

P1: “It serves for one family’s benefit.”

P2: “That family does not care about their own sanitation and hygiene.”

P3: “We can help in anything, but not latrine […] They would say ‘They poop by themselves so why do they need others to build latrine for them?’”

P1: “If they get sick or [go to the] hospital, we can send them to hospital.”

(FGD with men, Kandal province)

Strong local leadership was the only exception to the general lack of willingness to contribute to households that were unable to afford a latrine. Village leadership played an integral role in mobilizing resources within the community and linking villagers to resources outside of the community.

I: “Do you believe people in this village have ability to solve communal problems?”

P: “It depends on the village chief. If village chief made announcement about the development plan in this village such as building or fixing the road, people contribute their money to help.”

I: “Without village chief taking lead in the activity, do you think people can come up on their own and solve the problem?”

P: “[…] it depends on the village chief.”

I: “Why?”

P: “It’s because village chief gets money from any NGO that works in this village […] They depend on village chief and commune chief. If they need anything, they make suggestion to local authorities.”

(Village chief, age 70, man, Takeo province)

“I do not know where to find help from outside. I have to ask the village chief.”

(Key informant, age 49, man, Kampong Speu province)

This mobilization and convening role of local leadership, however, is seen as virtually exclusive to formal local leaders. Strong norms in favor of this hierarchical structure of leadership limit bottom-up decision making even within the government hierarchy and may explain the very limited number and role of organically formed community groups.

I: “If they had their own idea for improvement, would they be able to do something about it? What is the process for that?”

P2: “We have to inform village chief first if there is any problem that needs to be solved. We dare not to solve by ourselves.”

P6: “[…] we have to report to our leader first and discuss how we can solve this problem.”

I: “Why do you have to tell the village chief?”

P2: “It’s because we are the village members, so we dare not to make decisions by ourselves […] We should leave it to our leader because they have status.”

(FGD with man, Kampong Speu)

#### 3.2.4. Motivational Investment

Motivational investment was also found to be an important influencer of CE appraisals. During KIIs and FGDs, participants were asked whether they believed their community had the ability to come together to achieve a communal goal. Participants who did not believe their communities had this ability often reported that they themselves or others in their community did not have the skills or knowledge needed to achieve communal goals. Some of these individuals indicated that strict hierarchies limited autonomy to initiate collective action. Others stated that expectations for material support from external sources contributed to lower levels of community agency.

However, village chiefs frequently reported that community members could be motivated to take part in collective efforts once they understood the risks and benefits of the cooperative activity. Local leaders explained that this kind of understanding could come from NGO trainings or from witnessing others in the community engaging in the behavior and achieving success or reaping benefits.

“They [villagers] have ability [to work together]. First, they understand about it and second, they know how to do it […] In the past years, they were supported by NGO. Now they can walk by themselves. First, they understand about the problem. Since they had attended various meetings they gain knowledge. They start to solve problem with the small one first and it becomes bigger and bigger now.”

(Commune councilor, age 61, woman, Kampot province)

### 3.3. CFA of Contextualized and Hypothesized CE Frameworks

#### 3.3.1. Model 1: Women, Contextualized CE Model

The four-factor contextualized CE model demonstrated adequate model fit when applied to data generated from women respondents (RMSEA = 0.052, 90% CI = 0.047–0.058); χ^2^:df ratio = 2.113; CFI = 0.884; TLI = 0.873) (Table 3). Fifteen items with factor loadings <0.30 were omitted. Factor 1 consisted of six items dealing with social control including items concerning social order, normative beliefs, and willingness to intervene. Factor 2 included nine items about social cohesion including items concerning social equity, community attachment, and solidarity. Factor 3 included five items about social capital including items concerning social networks, community groups, community leadership, and trust. Factor 4 included seven items about motivational investment including items concerning self-efficacy, agency, and fulfillment of goals/needs (Table S3).

Three items were included in the contextualized CE model for women, but not for men. These items concerned perceptions of the extent to which beliefs about right and wrong are shared among the community (CE5), whether bribing community leaders is necessary for action (CE11), and whether neighbors would come together to help in the case of an unfortunate event (CA9).

#### 3.3.2. Model 2: Men, Contextualized CE Model

The four-factor, contextualized model demonstrated adequate model fit when applied to data generated from men (RMSEA = 0.062, 90% CI = 0.054–0.070); χ^2^:df ratio = 1.720; CFI = 0.872; TLI = 0.861) (Table 3). Fourteen items with factor loadings <0.30 were omitted. Factor 1 included seven items dealing with social control including items concerning social order and willingness to intervene. Factor 2 included nine items about social cohesion including items concerning social equity, community attachment, and solidarity. Factor 3 included four items about social capital including items concerning social networks, community groups, community leadership, and trust. Factor 4 included eight items about motivational investment including items concerning self-efficacy, agency, and fulfillment of goals/needs (Table S3).

Four items were included in the contextualized CE model for men, but not for women. These items concerned perceptions about whether neighbors would contribute time or money to common development goals (CA7), or would be sanctioned for not participating in community activities (CA1) or for not owning a latrine (CA3). These also included an item concerning perceptions about the extent to which the community requires external assistance to make positive changes (CE20).

#### 3.3.3. Model 3: Women, Hypothesized CE Model

The hypothesized model, which was adapted, but not fully contextualized to the Cambodian context, utilized a three-factor solution. This three-factor model demonstrated adequate model fit when applied to data generated from women (RMSEA = 0.055, 90% CI = 0.050–0.060); χ^2^:df ratio = 2.243; CFI = 0.870; TLI = 0.858) (Table 3). Fifteen items with factor loadings <0.30 were omitted. Factor 1 included seven items dealing with social control including items concerning social order and social response to open defecation, latrine purchase/construction, and crime-like activities. Factor 2 included 13 items about social cohesion including items concerning social capital, social equity, community attachment, and common values. Factor 3 included seven items about agency/empowerment including items concerning self-efficacy, collective action, and response to obstacles (Table S4). The same three items that were included in the contextualized CE model for women only (CE5, CE11, CA9) were also included for women, but not for men in the hypothesized CE model.

#### 3.3.4. Model 4: Men, Hypothesized CE Model

This three-factor model demonstrated adequate model fit when applied to data generated from men (RMSEA = 0.067, 90% CI = 0.059–0.075); χ^2^:df ratio = 1.843; CFI = 0.863; TLI = 0.851) (Table 3). Fourteen items with factor loadings <0.30 were omitted. Factor 1 included eight items dealing with social control including items concerning social order and social response. Factor 2 included 12 items about social cohesion including items concerning social capital, social equity, community attachment, and common values. Factor 3 included eight items about agency/empowerment including items concerning self-efficacy, collective action, and response to obstacles (Table S4). The same four items that were included in the contextualized CE model for men only (CA1, CA3, CA7, CE20) were also included for men, but not for women in the hypothesized model.

### 3.4. Comparison of Model Fit: Contextualized vs. Hypothesized CE Models

Table 3 provides fit statistics for the four fitted models. While none of the models had a non-significant χ^2^, all four models had χ^2^:df ratios of less than 3:1. Chi-square model fit estimations are sensitive to sample size, which may have been the cause of the small *p*-values and apparent lack of fit using this method [35]. None of the models had RMSEA values less than or equal to 0.05; however, all four models had RMSEA values less than 0.08, and the contextualized CE model using data from women (Model 1) had the smallest RMSEA value (0.052). None of the models had CFI or TLI values greater than or equal to 0.90. The contextualized model using data from women (Model 1), however, had the largest CFI (0.884) and TLI values (0.873).

Fit statistics indicate that the contextualized CE model with data generated from women respondents (Model 1) was the best fitting model. When comparing models with data generated from women (Models 1 and 3), it is clear that the contextualized model demonstrated better fit than the hypothesized CE model. When comparing models with data generated from men (Models 2 and 4), again, the contextualized model demonstrated better fit than the hypothesized model (Table 3). See Table S1 in Supplementary Materials for all item distributions. 

## 4. Discussion

This study followed a concurrent triangulation mixed methods design to inductively develop a contextualized CE framework that reflects the rural Cambodian context based on qualitative inputs from CRSHIP target villages and compare this framework to an existing, hypothesized CE framework. CFA allowed for comparison of model fit statistics to evaluate construct validity of the two frameworks and determine the value added of qualitative formative research to fully contextualize surveys for quantitative measurement of collective efficacy.

### 4.1. Comparing CE Frameworks

All four models demonstrated adequate model fit according to absolute fit statistics, but comparative fit statistics were poor for all models. This indicates that the data fit the model reasonably well, but that there is likely another model that may fit the data better. This warrants exploratory factor analysis (EFA) to better identify the underlying structure of CE in rural Cambodia. The slightly better fit of the contextualized models suggests that some factors of CE generated by inputs provided by participants during qualitative research produced more appropriate, context-specific factor solutions and assessments of CE.

The hypothesized CE models for women (Model 3) and men (Model 4) were based on a framework that incorporated inputs from qualitative research in the Ethiopian and Indian contexts [23]. However, these hypothesized models were not informed by inputs from Cambodian participants, and they may, therefore, fail to account for some important sub-constructs of CE as it pertains to the rural Cambodian context. This may have contributed to the comparatively lower item–factor relationships in the hypothesized models (Table S4).

The contextualized CE models for women (Model 1) and men (Model 2) were based on a framework that incorporated inputs from CRSHIP village members, which represent perceptions specific to the rural Cambodian context. Therefore, the framework on which these models were based largely accounted for the additional, context-specific sub-constructs. However, the data used in the CFA came from items adapted from an existing instrument that was not specifically designed to capture data on each additional sub-construct identified in the qualitative work. For instance, perceived benefits, knowledge of risks or benefits of the given behavior, and action knowledge were conceptualized as sub-constructs of motivational investment in the contextualized CE framework. These sub-constructs were not measured in the existing instrument. Similarly, solidarity was a sub-construct of social cohesion in these models and consisted of common values/beliefs and shared needs/benefits. While some items in the existing instrument did query respondents about common values and beliefs, the instrument did not account for the influence of shared needs and benefits of collective action on CE. Finally, external accountability (e.g., to NGO staff) was identified in the qualitative findings as an important facet of social control but was not measured by any item in the existing instrument.

The contextualized CE framework included four factors (social control, social cohesion, social capital, and motivational investment), whereas the hypothesized framework included three factors (social capital, social cohesion, and agency/empowerment). Motivational investment consisted of agency, self-efficacy, knowledge and perceived benefit (Table 2). Conversely, the hypothesized framework elevated agency to the level of a factor with self-efficacy as one of its dimensions. Therefore, the existing CE instrument did not include any items that captured the additional, qualitatively derived dimensions of motivational investment (i.e., knowledge and perceived benefit).

Additionally, the hypothesized CE framework conceptualized social capital as a dimension of social cohesion. The qualitative work, however, indicated that social cohesion and social capital played distinct, but complementary, roles in influencing a community’s CE perceptions. Therefore, the contextualized CE models (Models 1 and 2) parsed out social capital from social cohesion. We posit that, in the study context, social capital refers to the social infrastructure of a community, whereas social cohesion refers to the bonding, attachment, and partiality that exist between the individuals and groups that make up that infrastructure. Yet, this parsing out of social capital from social cohesion is contrary to the majority of the published literature [22,40]. Thus, we see that the wholesale application of existing, theoretically grounded conceptualizations of CE would not be capable of capturing these contextual differences in social capital, social cohesion, and motivational investment. These findings illustrate the importance of conducting qualitative formative research that utilizes inductive methodologies to build contextualized frameworks.

While there were differences between the contextualized and hypothesized CE frameworks, it is important to recognize that all four models included social control, social capital, social cohesion, and agency in some capacity. Although the formative qualitative work yielded a more nuanced contextualized framework that was slightly better fitting than the existing, hypothesized framework, the frameworks did have several important similarities. Based on our study findings and the strength of existing CE tools [23], we advocate for the use of existing, theoretically grounded CE frameworks as a foundation upon which to build contextualized measurement tools. Importantly, tools that incorporate inputs from the types of communities in which they will be used are likely to yield not only better fitting mathematical models, but also more meaningful and more applicable assessments of community CE perceptions. More work is needed to determine which CE factors, domains, and dimensions transcend contexts.

### 4.2. Importance of Leadership

One sub-construct that emerged as an important factor of CE across multiple contexts was community leadership. A previous study conducted in rural and peri-urban Ethiopia examined CE using the same hypothesized CE framework along with additional qualitative methods [23]. The researchers employed EFA and CFA to test, refine, and assess the validity of the contextualized CE scale [23]. In the six-factor model of CE that resulted from that study, “community organization and leadership” emerged as an important factor of the social capital domain. The study also found that factor scores for two factors—social networks and personal agency—differed significantly between respondents with and without leadership roles in the community [23]. Preliminary findings from a study of CE in rural India, using the same hypothesized CE framework, also found leadership to be an important factor [19].

In rural Cambodia, the relationship between local leaders and their communities follows a “patron–client” model, where local leaders serve as patrons who provide protection and benefits to their community members. The community members reciprocate by extending their allegiance, support, and assistance [41]. Our evidence, generated from CRSHIP villages, indicated deeply embedded traditions of patron–client relationships. Local leaders provided social safety nets for community members, who reciprocated by contributing time, money, or support for community development projects. Qualitative findings from our study included many examples of local leaders working to mobilize human resources within their own communities and linking villagers to resources outside of the community. In this way, local leadership was an important component of social capital. Indeed, the existing literature concerning local leadership in Cambodia suggests that personal patronage, although viewed as a form of corruption by some, is often used to obtain funding for public services [42]. The patron–client model is also reflected within the government hierarchy. Our qualitative findings corroborate findings from literature which asserts that village chiefs and commune councilors are disincentivized from taking initiative unless higher levels of government offer direct guidance and/or approval [43,44,45]. Understanding this mechanism may be key to leveraging local leadership to facilitate collective action toward sanitation uptake in Cambodia.

While community leadership appears to play a key role in influencing CE appraisals across contexts, it would be important for practitioners to understand the nuanced mechanism by which leadership operates in their specific program setting in order to most effectively leverage local leadership to enhance CE and facilitate collective action.

### 4.3. Sanitation as a Public Good

We observed evidence of collective action in study villages; however, we found no evidence of individuals or families purchasing or constructing a latrine for households in their village that were otherwise unable to afford one. As indicated above, we found strong and pervasive norms in favor of community members contributing time or money for village infrastructure, celebration of village ceremonies, and neighbors in urgent need. However, these socially embedded practices did not apply to sanitation. When asked if they would be willing to provide financial assistance to a neighbor for the construction or purchase of a latrine, almost all participants stated that they were unwilling to do so. Thus, it is not that there is low CE in these communities necessarily, but rather that the indigenous mechanisms that facilitate collective action in these communities are not perceived to be appropriate for use in increasing sanitation coverage. Therefore, our results suggest that sanitation was not perceived as a public good in these rural Cambodian study villages.

Many of the approaches used by local partners in implementing CRSHIP assume that communities will come together to build or buy latrines for those households in their village who are unable to do so. Our findings suggest that programs with theories of change that depend upon this form of collective action for reaching ODF status or 100% sanitation coverage should assess the extent to which sanitation is perceived to be a public good in target communities prior to implementation. If sanitation is not naturally perceived of in this way, program implementers should either (i) select a more appropriate program design that is not predicated on this assumption, or (ii) incorporate specific intervention techniques that are designed to shift these perceptions [23]. For instance, program implementers can work to improve the understanding that having access to and exclusively using an improved sanitation facility fulfills a relational obligation that people in a community have to each other in terms of their contributions to a common interest (e.g., open defecation free status, sanitary community environment). Alternatively, program implementers can require community-level sanitation (e.g., predetermined coverage level and exclusive use) prior to working with a community to install or develop communal infrastructure that is endogenously perceived of as a public good, such as a community water source or water distribution system [46].

Additionally, practitioners should assess CE perceptions prior to implementing community-based interventions that are contingent upon collective action. If CE appraisals are weak, practitioners will need to either incorporate intervention techniques that aim to strengthen CE appraisals [23], or identify program designs that do not require collective action.

### 4.4. Limitations and Strengths

This study had several strengths and limitations. The findings reflect the perceptions of adults in rural villages of Cambodia that were triggered by CRSHIP; they cannot necessarily be generalized to other populations. This limits the external validity of study findings. While two analysts conducted open coding and co-constructed the codebook, only one analyst applied codes across all transcripts. This is a limitation of our study in that it may have resulted in biased coding. The concurrent triangulation mixed methods study design and modified grounded theory approach were strengths of the study, and they contributed greatly to the internal validity of study findings. While the existing hypothesized CE framework performed relatively well, differences between the hypothesized and contextualized CE frameworks demonstrate the importance of inductive and iterative methodologies for the development of context-specific CE frameworks.

The existing CE survey was not used in its entirety. The 20 items that were dropped from the original 50-item survey were replaced with 12 items modified from the World Bank’s Integrated Questionnaire for the Measurement of Social Capital. As such, the 42 items had differing response options (Table S2), which likely had implications for model fit.

There are various recommendations for minimum CFA sample sizes. Some rely on the absolute number of respondents; these recommendations range from 150 to 300 [47,48]. Others rely on respondent-to-variable ratio; these recommendations range from 5:1 to 10:1 [48]. The intention was to obtain a sample of approximately 300 men and 300 women; however, with a 42-item instrument and a sample of 410 women and 186 men, the actual sample fell just short of the lower range of a sufficient sample size for men. The smaller sample size resulted in computational difficulties when fitting the CE models with data generated from men. These difficulties may have influenced the results of our construct validity assessments. The size of our overall sample also prevented us from conducting a subsequent EFA after observing that the hypothesized and contextualized CE models yielded relatively low comparative model fit statistics. Subsequent exploratory analyses would have required further partitioning of the data to create separate datasets for EFA and CFA, which was not possible due to the sample size. Therefore, the underlying factor structure, as derived from the quantitative data, was not identified during this study.

## 5. Conclusions

This study contributes to the small, yet growing body of literature in which CE is examined in the context of public health programs, particularly those that include community-based WASH interventions. This study offers both qualitative and quantitative evidence to demonstrate that the underlying structures through which CE functions appear to have key similarities across contexts and important differences between contexts. As demonstrated through this study, qualitative formative research that employs inductive methodologies is valuable for the development of fully contextualized CE measurement tools. Generalized CE frameworks and existing scales provide a foundation upon which researchers can build contextually appropriate measurement tools that incorporate inputs from community members. Comparisons of multiple, context-specific CE scales can then facilitate the identification of common CE factors and allow for global assessments of the set of common CE factors that transcend context [49].

Our findings have important consequences for program design, targeting, and evaluation. Public health programs that require collective action rely on two critical assumptions: (i) target participants believe that their community has the ability and autonomy to take action toward collective goals; and (ii) target participants agree that program goals reflect common interests that warrant collective action. As evidenced by our study findings, these are high-risk assumptions that must be assessed prior to program implementation. These assumptions should also be accounted for explicitly in program design, targeting, and evaluation. Failure to do so may result in ineffective community-based public health and development programs, as well as evaluation data that do not properly identify the factors contributing to program shortcomings.

## Figures and Tables

**Table 1:** Key informant interview (KII)/focus group discussion (FGD) participant demographics and survey respondent demographics, by sex.

	**KII/FGD Participants**			**Household Survey Respondents**		
	**Aggregate (*N*)**	**Women *n* (%)**	**Men *n* (%)**	**Aggregate *N***	**Women *n* (%)**	**Men *n* (%)**
Village clusters	**7**			30		
Households				596		
Respondents	114	52 (46)	62 (54)	596	410 (69)	186 (31)
**Respondent Demographics**						
**Relation to Head of Household**						
Self				303 (51)	147 (34)	156 (84)
Spouse				222 (37)	205 (50)	17 (9.1)
Sister/Brother				7 (1.2)	6 (1.5)	1 (0.54)
Daughter/Son				38 (6.4)	31 (7.6)	7 (3.8)
Mother/Father				26 (4.4)	21 (5.1)	5 (2.7)
**Household-Level Characteristics**						
Wealth Indicator *						
ID Poor 1	14 (13)	8 (16)	6 (10)	74 (12)	61 (15)	13 (7)
ID Poor 2	18 (17)	12 (24)	6 (10)	84 (14)	58 (14)	26 (14)
Not ID Poor	77 (71)	30 (60)	47 (80)	438 (73)	291 (71)	147 (79)
**Number of Members Per Household**						
Median (IQR) **^†^**	5 (4–6)	5 (4–6)	5 (4–6)	4 (4–5)	4 (4–5)	4 (3–5)
**Age of Head of Household**						
Median (IQR) **^†^**	54 (39–63)	52 (37.5–60.5)	55 (39–65)	47 (37–56)	47 (37–56)	46 (37–56)
**Head of Household’s Education**						
None	18 (16)	14 (27)	4 (6.5)	114 (19)	90 (22)	24 (13)
Primary	63 (55)	26 (50)	37 (60)	262 (44)	177 (43)	85 (46)
Secondary	25 (22)	8 (15)	17 (27)	149 (25)	103 (25)	46 (25)
High School	8 (7.0)	4 (7.7)	4 (6.5)	66 (11)	35 (8.5)	31 (17)
University	0 (0)	0 (0)	0 (0)	5 (0.8)	5 (1.2)	0 (0)
**Household Latrine Ownership ^‡^**						
Yes	85 (75)	31 (60)	54 (89)	317 (53)	206 (50)	111 (60)
No	28 (25)	21 (40)	7 (11)	279 (47)	204 (50)	75 (40)

* Classification according to the Identification of Poor Households Program of the Royal Government of Cambodia’s Ministry of Planning (ID Poor 1 considered “very poor,” ID Poor 2 considered “poor”), 5 KII/FGD participants with missing data; **^†^** IQR—interquartile range; ^‡^ Confirmed visually whenever possible, 1 KII/FGD participant with missing data.

**Table 2:** Contextualized framework for collective efficacy (CE) with inputs from rural Cambodian contexts.

Domains	Dimensions	Definition of Dimension	Examples of Associated Facets
Social control	Social order	Degree to which the community exists harmoniously as well as the presence or absence of crime and crime-like activities	Crime, crime-like activities
	Normative beliefs *	Unspoken or embedded community “rules” about the kinds of behaviors that are or are not socially acceptable and trigger sanctions, including positive reinforcements	Community norms, rules
	Intervention	Willingness and tendency for family, neighbors, community leaders to intervene when someone in the community engages in “undesired” behavior, or to reinforce “desired” behavior	Interpersonal/informal intervention, formal community sanctions, external accountability
Social cohesion	Social equity	Distribution of resources and opportunities within the community and the degree to which this distribution does or does not favor certain people, families, or groups within the community	Distribution of resources, contribution of resources, power
	Solidarity	Degree to which members of the community perceive themselves to be aligned with the group and the tendency of community members to act in this group’s interest	Common values/beliefs, shared needs/benefits, dependency
	Community attachment	Degree to which members of the community feel a sense of belonging with or proclivity for their community itself and other members of their community	Partiality, discrimination, belonging
Social capital	Social networks	Social network ties between family and neighbors in the village that facilitate the dissemination of knowledge, ideas, and social support	Communication, information sharing
	Community groups	Organizations, committees, or interest groups that have active membership in the village	Organic/social groups, community associations
	Community leadership	Formal or semi-formal leaders that work directly with the community; these include village chiefs, sub-village chiefs, commune councilors, religious leaders, and leaders of village committees and organizations	Linking networks to NGOs/external sources, government networks
	Trust	Perceptions about the reliability of the contacts in one’s familial and community networks, as well as the reliability of individuals and institutions outside of one’s networks	Endogenous trust, exogenous trust
Motivational investment	Self-efficacy	Individual community members’ beliefs about their capability to contribute to a community development project or cooperate and organize with other community members	Access to resources, mastery experience
	Agency	Beliefs about one’s own or one’s community’s control over one’s surroundings and fate	Power to act, locus of control
	Knowledge	Knowledge of the risks and benefits of engaging or not engaging in certain activities or behaviors, “how to” or action knowledge including skills needed to carry out the given behavior or activity	Knowledge of risks/benefits, “how to” knowledge
	Perceived benefit	Degree to which individuals believe they or their community stand to benefit from engaging in proposed collective action	Fulfillment of goal/needs, provision of incentive

* We recognize that there are aspects of normative beliefs that feed into both social control and motivational investment; however, for the purposes of this work, we conceptualized normative beliefs as a sub-construct of social control.

**Table 3:** Model fit statistics. CE—collective efficacy; RMSEA—root-mean-square error of approximation; CFI—comparative fit index; TLI—Tucker–Lewis index.

	Model 1	Model 2	Model 3	Model 4
Absolute Fit Statistics	Contextualized CE Model—Women	Contextualized CE Model—Men	Hypothesized CE Model—Women	Hypothesized CE Model—Men
χ^2^	676.276	643.291	719.975	643.282
Degrees of freedom (df)	320	374	321	349
χ^2^:df ratio	2.113	1.720	2.243	1.843
*p*-Value for χ^2^ test of model fit	*p* < 0.001	*p* < 0.001	*p* < 0.001	*p* < 0.001
RMSEA (90% confidence interval)	0.052 (0.047–0.058)	0.062 (0.054–0.070)	0.055 (0.050–0.060)	0.067 (0.059–0.075)
**Relative fit statistics**				
CFI	0.884	0.872	0.870	0.863
TLI	0.873	0.861	0.858	0.851

Estimation method: Weighted least squares with mean and variance adjustment (WLSMV) with sandwich estimator to adjust for non-independence of observations within 30 village clusters. Matrix: polychoric correlations.

## Supplementary Materials

The following are available online at https://www.mdpi.com/1660-4601/17/1/1/s1: Table S1: Item distributions: frequency of responses, by gender; Table S2: CE Tool; Table S3: Factor Loadings for Contextualized CE Models, by Gender of Respondent; Table S4. Factor Loadings for Hypothesized CE Models, by Gender of Respondent; File S1: Question guide for focus group discussions; File S2: Question guide for key informant interviews.
