# Supplementary material for: Sanitation and Collective Efficacy in Rural Cambodia: The Value Added of Qualitative Formative Work for the Contextualization of Measurement Tools
Source: Int J Environ Res Public Health. 2019 Dec 18;17(1):1. doi: 10.3390/ijerph17010001 (PMC6981916; doi:10.3390/ijerph17010001)
Supplement: Supplementary file 1 [file ijerph-17-00001-s001.pdf]

Supplementary Material

Table S1. Item Distributions: Frequency of responses, by gender.

Item response	Aggregate N = 596		Women n_W = 410		Men n_M = 186	
CA1. How likely is it that people who do not participate in community activities will be criticized or sanctioned by others in the community?						
Definitely not	99	16.61%	66	16.10%	33	17.74%
Probably not	74	12.42%	49	11.95%	25	13.44%
Uncertain	92	15.44%	67	16.34%	25	13.44%
Probably	178	29.87%	116	28.29%	62	33.33%
Definitely	153	25.67%	112	27.32%	41	22.04%
TOTAL	596, 100%		410, 100%		186, 100%	
CA3. How likely is it that people who do not own a latrine will be criticized or sanctioned by others in the community?						
Definitely not	34	5.70%	30	7.32%	4	2.15%
Probably not	23	3.86%	21	5.12%	2	1.08%
Uncertain	36	6.04%	25	6.10%	11	5.91%
Probably	196	32.89%	118	28.78%	78	41.94%
Definitely	307	51.51%	216	52.68%	91	48.92%
TOTAL	596, 100%		410, 100%		186, 100%	
CA5. How likely is it that people who do not send their children to school will be criticized or sanctioned by others in the community?						
Definitely not	21	3.52%	18	4.39%	3	1.61%
Probably not	8	1.34%	7	1.71%	1	0.54%
Uncertain	21	3.52%	17	4.15%	4	2.15%
Probably	154	25.84%	109	26.59%	45	24.19%
Definitely	392	65.77%	259	63.17%	133	71.51%
TOTAL	596, 100%		410, 100%		186, 100%	
CA7. What proportion of people in this village/community contribute time or money toward common development goals, such as building a well or repairing a road?						
No one	32	5.37%	24	5.85%	8	4.30%
Less than half	103	17.28%	75	18.29%	28	15.05%
About half	68	11.41%	52	12.68%	16	8.60%
More than half	155	26.01%	105	25.61%	50	26.88%
Everyone	238	39.93%	154	37.56%	84	45.16%
TOTAL	596, 100%		410, 100%		186, 100%	

CA8. If there was a water supply problem in this village/community how likely is it that people will cooperate to try to solve the problem?						
Definitely not	15	2.52%	13	3.17%	2	1.08%
Probably not	39	6.54%	24	5.85%	15	8.06%
Uncertain	59	9.90%	36	8.78%	23	12.37%
Probably	215	36.07%	150	36.59%	65	34.95%
Definitely	268	44.97%	187	45.61%	81	43.55%
TOTAL	596, 100%		410, 100%		186, 100%	
CA9. Suppose something unfortunate happened to someone in the village/community such as a serious illness, or the death of a parent. How likely is it that some people in the community would get together to help them?						
Definitely not	3	0.50%	3	0.73%	0	0
Probably not	2	0.34%	2	0.49%	0	0
Uncertain	6	1.01%	4	0.98%	2	1.08%
Probably	89	14.93%	60	14.63%	29	15.59%
Definitely	496	83.22%	341	83.17%	155	83.33%
TOTAL	596, 100%		410, 100%		186, 100%	
CE1. Most people in this community have common values, for example, they value hard work.						
Disagree to the greatest extent	8	1.34%	4	0.98%	4	2.15%
Somewhat disagree	31	5.20%	24	5.85%	7	3.76%
Neither agree nor disagree	155	26.01%	91	22.20%	64	34.41%
Somewhat agree	168	28.19%	110	26.83%	58	31.18%
Agree to the greatest extent	234	39.26%	181	44.15%	53	28.49%
TOTAL	596, 100%		410, 100%		186, 100%	
CE2. People in this community live in harmony with each other most of the time.						
Disagree to the greatest extent	12	12.01%	9	2.20%	3	1.61%
Somewhat disagree	31	5.20%	22	5.37%	9	4.84%
Neither agree nor disagree	131	21.98%	84	20.49%	47	25.27%
Somewhat agree	184	30.87%	119	29.02%	65	34.95%
Agree to the greatest extent	238	39.93%	176	42.93%	62	33.33%
TOTAL	596, 100%		410, 100%		186, 100%	
CE3. In this community, you have to be careful, otherwise your neighbors may cheat you.						
Disagree to the greatest extent	80	13.42%	54	13.17%	26	13.98%
Somewhat disagree	85	14.26%	50	12.20%	35	18.82%
Neither agree nor disagree	125	20.97%	79	19.27%	46	24.73%
Somewhat agree	180	30.20%	131	31.95%	49	26.34%
Agree to the greatest extent	126	21.14	96	23.41%	30	16.13%

TOTAL	596, 100%		410, 100%		186, 100%	
CE4. In this community, conflicts like stealing and fighting often occur.						
Disagree to the greatest extent	116	19.46%	77	18.78%	39	20.97%
Somewhat disagree	105	17.62%	66	16.10%	39	20.97%
Neither agree nor disagree	157	26.34%	97	23.66%	60	32.26%
Somewhat agree	189	31.71%	142	34.63%	47	25.27%
Agree to the greatest extent	29	4.87%	28	6.83%	1	0.54%
TOTAL	596, 100%		410, 100%		186, 100%	
CE5. Most people in this community have similar beliefs about what is right and what is wrong.						
Disagree to the greatest extent	19	3.19%	13	3.17%	6	3.23%
Somewhat disagree	47	7.89%	30	7.32%	17	9.14%
Neither agree nor disagree	233	39.09%	148	36.10%	85	45.70%
Somewhat agree	240	40.27%	174	42.44%	66	35.48%
Agree to the greatest extent	57	9.56%	45	10.98%	12	6.45%
TOTAL	596, 100%		410, 100%		186, 100%	
CE6. If the people of this community see crime-like activities, they will do something about it.						
Disagree to the greatest extent	5	0.84%	3	0.73%	2	1.08%
Somewhat disagree	9	1.51%	8	1.95%	1	0.54%
Neither agree nor disagree	74	12.42%	40	9.76%	34	18.28%
Somewhat agree	244	40.94%	172	41.95%	72	38.71%
Agree to the greatest extent	264	44.30%	187	45.61%	77	41.40%
TOTAL	596, 100%		410, 100%		186, 100%	
CE7. If there is a big dispute between two persons, other people from the community will help in solving the problem.						
Disagree to the greatest extent	22	3.69%	16	3.90%	6	3.23%
Somewhat disagree	12	2.01%	9	2.20%	3	1.61%
Neither agree nor disagree	96	16.11%	66	16.10%	30	16.13%
Somewhat agree	277	46.48%	190	46.34%	87	46.77%
Agree to the greatest extent	189	31.71%	129	31.46%	60	32.26%
TOTAL	596, 100%		410, 100%		186, 100%	
CE8. Differences between people, such as the amount of land they own, often causes problems in this community.						
Disagree to the greatest extent	131	21.98%	81	19.76%	50	26.88%
Somewhat disagree	104	17.45%	67	16.34%	37	19.89%
Neither agree nor disagree	178	29.87%	118	28.78%	60	32.26%
Somewhat agree	150	25.17%	111	27.07%	39	20.97%

Agree to the greatest extent	33	5.54%	33	8.05%	0	0
TOTAL	596, 100%		410, 100%		186, 100%	
CE9. People in this community praise households for installing a latrine.						
Disagree to the greatest extent	6	1.01%	6	1.46%	0	0
Somewhat disagree	11	1.85%	8	1.95%	3	1.61%
Neither agree nor disagree	68	11.41%	45	10.98%	23	12.37%
Somewhat agree	257	43.12%	169	41.22%	88	47.31%
Agree to the greatest extent	254	42.62%	182	44.39%	72	38.71%
TOTAL	596, 100%		410, 100%		186, 100%	
CE10. When community leaders make decisions, they are pleasing and good for most of the households in this community.						
Disagree to the greatest extent	12	2.01%	9	2.20%	3	1.61%
Somewhat disagree	21	3.52%	16	3.90%	5	2.69%
Neither agree nor disagree	162	27.18%	96	23.41%	66	35.48%
Somewhat agree	216	36.24%	156	38.05%	60	32.26%
Agree to the greatest extent	185	31.04%	133	32.44%	52	27.96%
TOTAL	596, 100%		410, 100%		186, 100%	
CE11. Sometimes people need to bribe community leaders in order to get things done. Read response options.						
Disagree to the greatest extent	159	26.68%	110	26.83%	49	26.34%
Somewhat disagree	67	11.24%	36	8.78%	31	16.67%
Neither agree nor disagree	182	30.54%	119	29.02%	63	33.87%
Somewhat agree	150	25.17%	116	28.29%	34	18.28%
Agree to the greatest extent	38	6.38%	29	7.07%	9	4.84%
TOTAL	596, 100%		410, 100%		186, 100%	
CE12. During a crisis situation, such as a drought, flood, or a fire, government services are distributed equally by the community to all households in need.						
Disagree to the greatest extent	54	9.06%	36	8.78%	18	9.68%
Somewhat disagree	51	8.56%	30	7.32%	21	11.29%
Neither agree nor disagree	155	26.01%	104	25.37%	51	27.42%
Somewhat agree	209	35.07%	153	37.32%	56	30.11%
Agree to the greatest extent	127	21.31%	87	21.22%	40	21.51%
TOTAL	596, 100%		410, 100%		186, 100%	
CE13. Some households in this community are restricted from receiving NGO/civil society services, such as agricultural assistance.						
Disagree to the greatest extent	222	37.25%	144	35.12%	78	41.94%
Somewhat disagree	78	13.09%	50	12.20%	28	15.05%
Neither agree nor disagree	140	23.49%	99	24.15%	41	22.04%

Somewhat agree	129	21.64%	98	23.90%	31	16.67%
Agree to the greatest extent	27	4.53%	19	4.63%	8	4.30%
TOTAL	596, 100%		410, 100%		186, 100%	
	CE14. People in this community accept me as a member of the community.					
Disagree to the greatest extent	0	0	0	0	0	0
Somewhat disagree	4	0.67%	3	0.73%	1	0.54%
Neither agree nor disagree	45	7.55%	27	6.59%	18	9.68%
Somewhat agree	183	30.70%	119	29.02%	64	34.41%
Agree to the greatest extent	364	61.07%	261	63.66%	103	55.38%
TOTAL	596, 100%		410, 100%		186, 100%	
	CE15. I feel attached to this community and its people.					
Disagree to the greatest extent	3	0.50%	2	0.49%	1	0.54%
Somewhat disagree	0	0	0	0	0	0
Neither agree nor disagree	70	11.74%	36	8.78%	34	18.28%
Somewhat agree	193	32.28%	141	34.39%	52	27.96%
Agree to the greatest extent	330	55.37%	231	56.34%	99	53.23%
TOTAL	596, 100%		410, 100%		186, 100%	
	CE16. I feel proud to be part of this community.					
Disagree to the greatest extent	2	0.34%	1	0.24%	1	0.54%
Somewhat disagree	4	0.67%	2	0.49%	2	1.08%
Neither agree nor disagree	87	14.60%	47	11.46%	40	21.51%
Somewhat agree	169	28.36%	124	30.24%	45	24.19%
Agree to the greatest extent	334	56.04%	236	57.56%	98	52.69%
TOTAL	596, 100%		410, 100%		186, 100%	
	CE17. I have the capacity to achieve my future aims.					
Disagree to the greatest extent	48	8.05%	36	8.78%	12	6.45%
Somewhat disagree	38	6.38%	22	5.37%	16	8.60%
Neither agree nor disagree	149	25.00%	94	22.93%	55	29.57%
Somewhat agree	202	33.89%	146	35.61%	56	30.11%
Agree to the greatest extent	159	26.68%	112	27.32%	47	25.27%
TOTAL	596, 100%		410, 100%		186, 100%	
	CE18. I have the ability to contribute to this community's development.					
Disagree to the greatest extent	13	2.18%	10	2.44%	3	1.61%
Somewhat disagree	54	9.06%	32	7.80%	22	11.83%

Neither agree nor disagree	171	28.69%	111	27.07%	60	32.26%
Somewhat agree	261	43.79%	192	46.83%	69	37.10%
Agree to the greatest extent	97	16.28%	65	15.85%	32	17.20%
TOTAL	596, 100%		410, 100%		186, 100%	
	CE19. People in this community have the capacity to make positive changes by coming together.					
Disagree to the greatest extent	2	0.34%	2	0.49%	0	0
Somewhat disagree	10	1.68%	6	1.46%	4	2.15%
Neither agree nor disagree	119	19.97%	75	18.29%	44	23.66%
Somewhat agree	343	57.55%	236	57.56%	107	57.53%
Agree to the greatest extent	122	20.47%	91	22.20%	31	16.67%
TOTAL	596, 100%		410, 100%		186, 100%	
	CE20. This community needs assistance from others outside the community in order to make positive changes.					
Disagree to the greatest extent	4	0.67%	2	0.49%	2	1.08%
Somewhat disagree	13	2.18%	8	1.95%	5	2.69%
Neither agree nor disagree	73	12.25%	46	11.22%	27	14.52%
Somewhat agree	265	44.46%	177	43.17%	88	47.31%
Agree to the greatest extent	241	40.44%	177	43.17%	64	34.41%
TOTAL	596, 100%		410, 100%		186, 100%	
	CE21. People in this community should work together to develop the community.					
Disagree to the greatest extent	1	0.17%	0	0	1	0.54%
Somewhat disagree	6	1.01%	2	0.49%	4	2.15%
Neither agree nor disagree	36	6.04%	30	7.32%	6	3.23%
Somewhat agree	249	41.78%	161	39.27%	88	47.31%
Agree to the greatest extent	304	51.01%	217	52.93%	87	46.77%
TOTAL	596, 100%		410, 100%		186, 100%	
	CE22. People in this community can be trusted.					
Disagree to the greatest extent	2	0.34%	1	0.24%	1	0.54%
Somewhat disagree	5	0.84%	2	0.49%	3	1.61%
Neither agree nor disagree	153	25.67%	104	25.37%	49	26.34%
Somewhat agree	263	44.13%	179	43.66%	84	45.16%
Agree to the greatest extent	173	29.03%	124	30.24%	49	26.34%
TOTAL	596, 100%		410, 100%		186, 100%	
	CE23. The leaders of community-based associations respond to this community's concerns.					
Disagree to the greatest extent	6	1.01%	4	0.98%	2	1.08%

Somewhat disagree	6	1.01%	5	1.22%	1	0.54%
Neither agree nor disagree	117	19.63%	77	18.78%	40	21.51%
Somewhat agree	262	43.96%	171	41.71%	91	48.92%
Agree to the greatest extent	205	34.40%	153	37.32%	52	27.96%
TOTAL	596, 100%		410, 100%		186, 100%	
	CE24. This community's leaders can be trusted.					
Disagree to the greatest extent	7	1.17%	6	1.46%	1	0.54%
Somewhat disagree	15	2.52%	8	1.95%	7	3.76%
Neither agree nor disagree	98	16.44%	68	16.59%	30	16.13%
Somewhat agree	266	44.63%	177	43.17%	89	47.85%
Agree to the greatest extent	210	35.23%	151	36.83	59	31.72%
TOTAL	596, 100%		410, 100%		186, 100%	
	CE25. People in this community get to choose their local leaders.					
Disagree to the greatest extent	3	0.50%	2	0.49%	1	0.54%
Somewhat disagree	4	0.67%	4	0.98%	0	0
Neither agree nor disagree	52	8.72%	33	8.05%	19	10.22%
Somewhat agree	183	30.70%	137	33.41%	46	24.73%
Agree to the greatest extent	354	59.40%	234	57.07%	120	64.52%
TOTAL	596, 100%		410, 100%		186, 100%	
	CE26. In this community, people prioritize their own family's welfare over community development.					
Disagree to the greatest extent	0	0	0	0	0	0
Somewhat disagree	7	1.17%	2	0.49%	5	2.69%
Neither agree nor disagree	126	21.14%	74	18.05%	52	27.96%
Somewhat agree	171	28.69%	119	29.02%	52	27.96%
Agree to the greatest extent	292	48.99%	215	52.44%	77	41.40%
TOTAL	596, 100%		410, 100%		186, 100%	
	CE27. Most people in this community have similar hopes about the future development of the community.					
Disagree to the greatest extent	2	0.34%	1	0.24%	1	0.54%
Somewhat disagree	12	2.01%	7	1.71%	5	2.69%
Neither agree nor disagree	151	25.34%	90	21.95%	61	32.80%
Somewhat agree	307	51.51%	224	54.63%	83	44.62%
Agree to the greatest extent	124	20.81%	88	21.46%	36	19.35%
TOTAL	596, 100%		410, 100%		186, 100%	
	CE28. If people in this community saw someone openly defecating, they would do or say something about it.					

Disagree to the greatest extent	14	2.35%	9	2.20%	5	2.96%
Somewhat disagree	12	2.01%	7	1.71%	5	2.96%
Neither agree nor disagree	85	14.26%	61	14.88%	24	12.90%
Somewhat agree	220	36.91%	141	34.39%	79	42.47%
Agree to the greatest extent	265	44.46%	192	46.83%	73	39.25%
TOTAL	596, 100%		410, 100%		186, 100%	
CE29. We, as a community, can overcome obstacles that we encounter when working toward a common goal.						
Disagree to the greatest extent	4	0.67%	1	0.24%	3	1.61%
Somewhat disagree	9	1.51%	4	0.98%	5	2.69%
Neither agree nor disagree	119	19.97%	72	17.56%	47	25.27%
Somewhat agree	326	54.70%	235	57.32%	91	48.92%
Agree to the greatest extent	138	23.15%	98	23.90%	40	21.51%
TOTAL	596, 100%		410, 100%		186, 100%	
CE30. People in this community are motivated to achieve common development goals, even when those goals seem challenging.						
Disagree to the greatest extent	9	1.51%	4	0.98%	5	2.69%
Somewhat disagree	14	2.35%	9	2.20%	5	2.69%
Neither agree nor disagree	107	17.95%	61	14.88%	46	24.73%
Somewhat agree	301	50.50%	228	55.61%	73	39.25%
Agree to the greatest extent	165	27.68%	108	26.34%	57	30.65%
TOTAL	596, 100%		410, 100%		186, 100%	
Netb2. If you suddenly needed a small amount of money [enough to pay for expenses for your household for one week], how many people beyond your immediate household could you turn to who would be willing to provide this money?						
No one	77	12.92%	59	14.39%	18	9.68%
One or two people	216	36.24%	157	38.29%	59	31.72%
Three or four people	162	27.18%	111	27.07%	51	27.42%
Five or more people	141	23.66%	83	20.24%	58	31.18%
TOTAL	596, 100%		410, 100%		186, 100%	
Netb4. If you suddenly had to go away for a day or two, could you count on relatives to take care of your children?						
Definitely not	55	9.23%	46	11.22%	9	4.84%
Probably not	23	3.86%	18	4.39%	5	2.69%
Uncertain	10	1.68%	6	1.46%	4	2.15%
Probably	80	13.42%	56	13.66%	24	12.90%
Definitely	428	71.81%	284	69.27%	144	77.42%
TOTAL	596, 100%		410, 100%		186, 100%	
Netb5. If you suddenly had to go away for a day or two, could you count on friends to take care of your children?						

Definitely not	296	49.66%	214	52.20%	82	44.09%
Probably not	119	19.97%	84	20.49%	35	18.82%
Uncertain	86	14.43%	54	13.17%	32	17.20%
Probably	57	9.56%	31	7.56%	26	13.98%
Definitely	38	6.38	27	6.59%	11	5.91%
TOTAL	596, 100%		410, 100%		186, 100%	
Netb6. If you suddenly had to go away for a day or two, could you count on neighbors to take care of your children?						
Definitely not	97	16.28%	79	19.27%	18	9.68%
Probably not	41	6.88%	35	8.54%	6	3.23%
Uncertain	53	8.89%	36	8.78%	17	9.14%
Probably	181	30.37%	118	28.78%	63	33.87%
Definitely	224	37.58%	142	34.63%	82	44.09%
TOTAL	596, 100%		410, 100%		186, 100%	
Netb7. If you suddenly faced a long-term emergency such as the death of family member or a natural disaster like drought, flood, or a fire, how many people beyond your immediate household could you turn to who would be willing to assist you?						
No one	121	20.30%	88	21.46%	33	17.74%
One or two people	134	22.48%	87	21.22%	47	25.27%
Three or four people	98	16.44%	65	15.85%	33	17.74%
Five or more people	243	40.77%	170	41.46%	73	39.25%
TOTAL	596, 100%		410, 100%		186, 100%	
Netb8. In the past 6 months, how many people with a personal problem have turned to you for assistance?						
No one	361	60.57%	258	62.93%	103	55.38%
One or two people	129	21.64%	75	18.29%	54	29.03%
Three or four people	72	12.08%	51	12.44%	21	11.29%
Five or more people	34	5.70%	26	6.34%	8	4.30%
TOTAL	596, 100%		410, 100%		186, 100%	

Table S2. CE Tool.

Item Name	Survey Item (i.e., Prompt)	Variable Values				
		1	2	3	4	5
CA1	How likely is it that people who do not participate in community activities will be criticized or sanctioned by others in the community?	Definitely not	Probably not	Uncertain	Probably	Definitely
CA3	How likely is it that people who do not own a latrine will be criticized or sanctioned by others in the community?	Definitely not	Probably not	Uncertain	Probably	Definitely
CA5 *	How likely is it that people who do not send their children to school will be criticized or sanctioned by others in the community?	Definitely not	Probably not	Uncertain	Probably	Definitely
CA7	What proportion of people in this village/community contribute time or money toward common development goals, such as building a well or repairing a road?	No one	Less than half	About half	More than half	Everyone
CA8	If there was a water supply problem in this village/community, how likely is it that people will cooperate to try to solve the problem?	Definitely not	Probably not	Uncertain	Probably	Definitely
CA9	Suppose something unfortunate happened to someone in the village/community such as a serious illness, or the death of a parent. How likely is it that some people in the community would get together to help them?	Definitely not	Probably not	Uncertain	Probably	Definitely
CE1	Most people in this community have common values, for example, they value hard work.	Disagree to the greatest extent	Somewhat disagree	Neither agree nor disagree	Somewhat agree	Agree to the greatest extent

CE2	People in this community live in harmony with each other most of the time.	Disagree to the greatest extent	Somewhat disagree	Neither agree nor disagree	Somewhat agree	Agree to the greatest extent
CE3	In this community, you have to be careful, otherwise your neighbors may cheat you.	Agree to the greatest extent	Somewhat agree	Neither agree nor disagree	Somewhat disagree	Disagree to the greatest extent
CE4 *	In this community, conflicts like stealing and fighting often occur.	Agree to the greatest extent	Somewhat agree	Neither agree nor disagree	Somewhat disagree	Disagree to the greatest extent
CE5	Most people in this community have similar beliefs about what is right and what is wrong.	Disagree to the greatest extent	Somewhat disagree	Neither agree nor disagree	Somewhat agree	Agree to the greatest extent
CE6	If the people of this community see crime-like activities, they will do something about it.	Disagree to the greatest extent	Somewhat disagree	Neither agree nor disagree	Somewhat agree	Agree to the greatest extent
CE7 *	If there is a big dispute between two persons, other people from the community will help in solving the problem.	Disagree to the greatest extent	Somewhat disagree	Neither agree nor disagree	Somewhat agree	Agree to the greatest extent
CE8 *	Differences between people, such as the amount of land they own, often cause problems in this community.	Agree to the greatest extent	Somewhat agree	Neither agree nor disagree	Somewhat disagree	Disagree to the greatest extent
CE9	People in this community praise households for installing a latrine.	Disagree to the greatest extent	Somewhat disagree	Neither agree nor disagree	Somewhat agree	Agree to the greatest extent
CE10	When community leaders make decisions, they are pleasing and good for most of the households in this community.	Disagree to the greatest extent	Somewhat disagree	Neither agree nor disagree	Somewhat agree	Agree to the greatest extent
CE11	Sometimes people need to bribe community leaders in order to get things done.	Agree to the greatest extent	Somewhat agree	Neither agree nor disagree	Somewhat disagree	Disagree to the greatest extent
CE12	During a crisis situation, such as a drought, flood, or a fire, government services are distributed equally by the community to all households in need.	Disagree to the greatest extent	Somewhat disagree	Neither agree nor disagree	Somewhat agree	Agree to the greatest extent

CE13 *	Some households in this community are restricted from receiving NGO/civil society services, such as agricultural assistance.	Agree to the greatest extent	Somewhat agree	Neither agree nor disagree	Somewhat disagree	Disagree to the greatest extent
CE14	People in this community accept me as a member of the community.	Disagree to the greatest extent	Somewhat disagree	Neither agree nor disagree	Somewhat agree	Agree to the greatest extent
CE15	I feel attached to this community and its people.	Disagree to the greatest extent	Somewhat disagree	Neither agree nor disagree	Somewhat agree	Agree to the greatest extent
CE16	I feel proud to be part of this community.	Disagree to the greatest extent	Somewhat disagree	Neither agree nor disagree	Somewhat agree	Agree to the greatest extent
CE17	I have the capacity to achieve my future aims.	Disagree to the greatest extent	Somewhat disagree	Neither agree nor disagree	Somewhat agree	Agree to the greatest extent
CE18	I have the ability to contribute to this community's development.	Disagree to the greatest extent	Somewhat disagree	Neither agree nor disagree	Somewhat agree	Agree to the greatest extent
CE19	People in this community have the capacity to make positive changes by coming together.	Disagree to the greatest extent	Somewhat disagree	Neither agree nor disagree	Somewhat agree	Agree to the greatest extent
CE20	This community needs assistance from others outside the community in order to make positive changes.	Agree to the greatest extent	Somewhat agree	Neither agree nor disagree	Somewhat disagree	Disagree to the greatest extent
CE21	People in this community should work together to develop the community.	Disagree to the greatest extent	Somewhat disagree	Neither agree nor disagree	Somewhat agree	Agree to the greatest extent
CE22	People in this community can be trusted.	Disagree to the greatest extent	Somewhat disagree	Neither agree nor disagree	Somewhat agree	Agree to the greatest extent
CE23	The leaders of community-based associations respond to this community's concerns.	Disagree to the greatest extent	Somewhat disagree	Neither agree nor disagree	Somewhat agree	Agree to the greatest extent
CE24	This community's leaders can be trusted.	Disagree to the greatest extent	Somewhat disagree	Neither agree nor disagree	Somewhat agree	Agree to the greatest extent
CE25	People in this community get to choose their local leaders.	Disagree to the greatest extent	Somewhat disagree	Neither agree nor disagree	Somewhat agree	Agree to the greatest extent
CE26	In this community, people prioritize their own family's welfare over community development.	Agree to the greatest extent	Somewhat agree	Neither agree nor disagree	Somewhat disagree	Disagree to the greatest extent

CE27	Most people in this community have similar hopes about the future development of the community.	Disagree to the greatest extent	Somewhat disagree	Neither agree nor disagree	Somewhat agree	Agree to the greatest extent
CE28	If people in this community saw someone openly defecating, they would do or say something about it.	Disagree to the greatest extent	Somewhat disagree	Neither agree nor disagree	Somewhat agree	Agree to the greatest extent
CE29	We, as a community, can overcome obstacles that we encounter when working toward a common goal.	Disagree to the greatest extent	Somewhat disagree	Neither agree nor disagree	Somewhat agree	Agree to the greatest extent
CE30	People in this community are motivated to achieve common development goals, even when those goals seem challenging.	Disagree to the greatest extent	Somewhat disagree	Neither agree nor disagree	Somewhat agree	Agree to the greatest extent
Netb2 *	If you suddenly needed a small amount of money [enough to pay for expenses for your household for one week], how many people beyond your immediate household could you turn to who would be willing to provide this money?	No one	One or two people	Three or four people	Five or more people	
Netb4 *	If you suddenly had to go away for a day or two, could you count on relatives to take care of your children?	Definitely not	Probably not	Uncertain	Probably	Definitely
Netb5 *	If you suddenly had to go away for a day or two, could you count on friends to take care of your children?	Definitely not	Probably not	Uncertain	Probably	Definitely
Netb6 *	If you suddenly had to go away for a day or two, could you count on neighbors to take care of your children?	Definitely not	Probably not	Uncertain	Probably	Definitely
Netb7 *	If you suddenly faced a long-term emergency such as the death of a family member or a natural disaster like drought, flood, or a fire, how many people beyond your	No one	One or two people	Three or four people	Five or more people	

	immediate household could you turn to who would be willing to assist you?					
Netb8 *	In the past 6 months, how many people with a personal problem had turned to you for assistance?	No one	One or two people	Three or four people	Five or more people	

* Items that were not included in the initial confirmatory factor analysis (CFA), but were removed as a result of item reduction and were not in the final CFA for any of the four models.

Table S3. Factor Loadings for Contextualized CE Models, by Gender of Respondent.

		Women (n_W = 410)		Men (n_M = 186)	
**Factors and Associated Items**	**Item**	**Initial CFA**	**Final CFA**	**Initial CFA**	**Final CFA**
**Factor 1:** ***Social Control***					
How likely is it that people who do not participate in community activities will be criticized or sanctioned by others in the community?	CA1	0.293 *	-	0.390 *	0.382 *
How likely is it that people who do not own a latrine will be criticized or sanctioned by others in the community?	CA3	0.337 *	-	0.532 *	0.525 *
How likely is it that people who do not send their children to school will be criticized or sanctioned by others in the community?	CA5	0.297 *	-	0.113	-
People in this community live in harmony with each other most of the time.	CE2	0.581 *	0.616 *	0.407 *	0.406 *
In this community, you have to be careful, otherwise your neighbors may cheat you.	CE3	−0.432 *	−0.418 *	−0.502 *	−0.469 *
In this community, conflicts like stealing and fighting often occur.	CE4	−0.036	-	0.084	-
Most people in this community have similar beliefs about what is right and what is wrong.	CE5	0.568 *	0.595 *	0.216 *	-
If the people of this community see crime-like activities, they will do something about it.	CE6	0.444 *	0.448 *	0.356 *	0.333 *
If there is a big dispute between two persons, other people from the community will help in solving the problem.	CE7	0.017	-	0.090	-
People in this community praise households for installing a latrine. Read response options.	CE9	0.615 *	0.611 *	0.654 *	0.652 *
If people in this community saw someone openly defecating, they would do or say something about it.	CE28	0.618 *	0.606 *	0.615 *	0.610 *
**Factor 2:** ***Social Cohesion***					
What proportion of people in this village/community contribute time or money toward common development goals, such as building a well or repairing a road?	CA7	−0.054	-	−0.473 *	−0.440 *

Most people in this community have common values, for example, they value hard work.	CE1	0.661 *	0.641 *	0.505 *	0.497 *
Differences between people, such as the amount of land they own, often cause problems in this community.	CE8	−0.155 *	-	-0.105	-
When community leaders make decisions, they are pleasing and good for most of the households in this community.	CE10	0.633 *	0.633 *	0.616 *	0.632 *
Sometimes people need to bribe community leaders in order to get things done.	CE11	0.365 *	0.380*	−0.142 *	-
During a crisis situation, such as a drought, flood, or a fire, government services are distributed equally by the community to all households in need	CE12	0.394 *	0.398 *	0.637 *	0.638 *
Some households in this community are restricted from receiving NGO/civil society services, such as agricultural assistance.	CE13	0.101 *	-	−0.275 *	-
People in this community accept me as a member of the community.	CE14	0.750 *	0.755 *	0.763 *	0.768 *
I feel attached to this community and its people.	CE15	0.750 *	0.758 *	0.775 *	0.773 *
I feel proud to be part of this community.	CE16	0.689 *	0.698 *	0.776 *	0.768 *
In this community, people prioritize their own family's welfare over community development.	CE26	−0.574 *	−0.564 *	−0.647 *	−0.646 *
Most people in this community have similar hopes about the future development of the community.	CE27	0.621 *	0.617 *	0.639 *	0.640 *
**Factor 3:** ***Social Capital***					
Suppose something unfortunate happened to someone in the village/community such as a serious illness, or the death of a parent. How likely is it that some people in the community would get together to help them?	CA9	0.427 *	0.418 *	−0.237 *	-
People in this community can be trusted. Read response options.	CE22	0.709 *	0.710 *	0.592 *	0.620 *
The leaders of community-based associations respond to this community's concerns.	CE23	0.911 *	0.914 *	0.772 *	0.804 *
This community's leaders can be trusted.	CE24	0.830 *	0.832 *	0.794 *	0.848 *
People in this community get to choose their local leaders.	CE25	0.492 *	0.481 *	0.475 *	0.537 *
If you suddenly needed a small amount of money [enough to pay for expenses for your household for one week], how many people beyond your immediate household could you turn to who would be willing to provide this money?	Netb2	−0.103 *	-	−0.327 *	-
If you suddenly had to go away for a day or two, could you count on relatives to take care of your children?	Netb4	0.047	-	−0.213 *	-
If you suddenly had to go away for a day or two, could you count on friends to take care of your children?	Netb5	0.022	-	−0.072	-

If you suddenly had to go away for a day or two, could you count on neighbors to take care of your children?	Netb6	−0.046	-	−0.286 *	-
If you suddenly faced a long-term emergency such as the death of a family member or a natural disaster like drought, flood, or a fire, how many people beyond your immediate household could you turn to who would be willing to assist you?	Netb7	−0.142 *	-	0.003	-
In the past 6 months, how many people with a personal problem had turned to you for assistance?	Netb8	0.098	-	−0.297 *	-
**Factor 4:** ***Motivational Investment***					
If there was a water supply problem in this village/community, how likely is it that people will cooperate to try to solve the problem?	CA8	0.308 *	0.306 *	0.409 *	0.386 *
I have the capacity to achieve my future aims.	CE17	0.579 *	0.591 *	0.455 *	0.448 *
I have the ability to contribute to this community's development.	CE18	0.380 *	0.383 *	0.654 *	0.646 *
People in this community have the capacity to make positive changes by coming together.	CE19	0.583 *	0.579 *	0.669 *	0.673 *
This community needs assistance from others outside the community in order to make positive changes.	CE20	−0.283 *	-	−0.439 *	−0.434 *
People in this community should work together to develop the community.	CE21	0.587 *	0.576 *	0.650 *	0.665 *
We, as a community, can overcome obstacles that we encounter when working toward a common goal.	CE29	0.799 *	0.769 *	0.666 *	0.672 *
People in this community are motivated to achieve common development goals, even when those goals seem challenging.	CE30	0.564 *	0.552 *	0.594 *	0.600 *

Estimation method: WLSMV with sandwich estimator to adjust for non-independence of observations within 30 village clusters. Matrix: polychoric correlations. * Statistically significant factor loadings at $p \leq 0.05$.

Table S4. Factor Loadings for Hypothesized CE Models, by Gender of Respondent.

		Women (n_W = 410)		Men (n_M = 186)	
**Factors and Associated Items**	**Item**	**Initial CFA**	**Final CFA**	**Initial CFA**	**Final CFA**
**Factor 1:** ***Social Control***					
How likely is it that people who do not participate in community activities will be criticized or sanctioned by others in the community?	CA1	0.289 *	-	0.385 *	0.407 *
How likely is it that people who do not own a latrine will be criticized or sanctioned by others in the community?	CA3	0.333 *	-	0.525 *	0.562 *

How likely is it that people who do not send their children to school will be criticized or sanctioned by others in the community?	CA5	0.294 *	-	0.108	-
What proportion of people in this village/community contribute time or money toward common development goals, such as building a well or repairing a road?	CA7	−0.057	-	−0.465 *	−0.458 *
Suppose something unfortunate happened to someone in the village/community such as a serious illness, or the death of a parent. How likely is it that some people in the community would get together to help them?	CA9	0.401 *	0.396 *	−0.207 *	-
People in this community live in harmony with each other most of the time.	CE2	0.581 *	0.612 *	0.405 *	0.427 *
In this community, you have to be careful, otherwise your neighbors may cheat you.	CE3	−0.424 *	−0.407 *	−0.496 *	−0.504 *
In this community, conflicts like stealing and fighting often occur.	CE4	−0.034	-	0.087	-
Most people in this community have similar beliefs about what is right and what is wrong.	CE5	0.563 *	0.585 *	0.214 *	-
If the people of this community see crime-like activities, they will do something about it.	CE6	0.437 *	0.439 *	0.352 *	0.415 *
If there is a big dispute between two persons, other people from the community will help in solving the problem.	CE7	0.016	-	0.089	-
Differences between people, such as the amount of land they own, often cause problems in this community.	CE8	−0.154 *	-	−0.091	-
People in this community praise households for installing a latrine.	CE9	0.609 *	0.601 *	0.645 *	0.697 *
If people in this community saw someone openly defecating, they would do or say something about it.	CE28	0.618 *	0.600 *	0.607 *	0.651 *
**Factor 2:** ***Social Cohesion***					
Most people in this community have common values, for example, they value hard work.	CE1	0.650 *	0.626 *	0.495 *	0.483 *
When community leaders make decisions, they are pleasing and good for most of the households in this community.	CE10	0.622 *	0.618 *	0.612 *	0.623 *
Sometimes people need to bribe community leaders in order to get things done.	CE11	0.373 *	0.383 *	−0.126	-
During a crisis situation, such as a drought, flood, or a fire, government services are distributed equally by the community to all households in need.	CE12	0.384 *	0.386 *	0.625 *	0.623 *
Some households in this community are restricted from receiving NGO/civil society services, such as agricultural assistance.	CE13	0.112 *	-	−0.257 *	-
People in this community accept me as a member of the community.	CE14	0.738 *	0.738 *	0.754 *	0.755 *
I feel attached to this community and its people.	CE15	0.734 *	0.738 *	0.765 *	0.760 *
I feel proud to be part of this community.	CE16	0.669 *	0.674 *	0.768 *	0.757 *
People in this community can be trusted.	CE22	0.664 *	0.667 *	0.509 *	0.522 *
The leaders of community-based associations respond to this community's concerns.	CE23	0.844 *	0.849 *	0.655 *	0.668 *
This community's leaders can be trusted.	CE24	0.809 *	0.813 *	0.687 *	0.709 *

People in this community get to choose their local leaders.	CE25	0.457 *	0.447 *	0.400 *	0.430 *
In this community, people prioritize their own family's welfare over community development.	CE26	−0.565 *	−0.552 *	−0.641 *	−0.635 *
Most people in this community have similar hopes about the future development of the community.	CE27	0.608 *	0.600 *	0.630 *	0.613 *
If you suddenly needed a small amount of money [enough to pay for expenses for your household for one week], how many people beyond your immediate household could you turn to who would be willing to provide this money?	Netb2	−0.098 *	-	−0.284 *	-
If you suddenly had to go away for a day or two, could you count on relatives to take care of your children?	Netb4	0.038	-	−0.182 *	-
If you suddenly had to go away for a day or two, could you count on friends to take care of your children?	Netb5	0.019	-	−0.054	-
If you suddenly had to go away for a day or two, could you count on neighbors to take care of your children?	Netb6	−0.050	-	−0.247 *	-
If you suddenly faced a long-term emergency such as the death of a family member or a natural disaster like drought, flood, or a fire, how many people beyond your immediate household could you turn to who would be willing to assist you?	Netb7	−0.122 *	-	0.027	-
In the past 6 months, how many people with a personal problem had turned to you for assistance?	Netb8	0.090	-	−0.242 *	-
**Factor 3:** ***Agency/Empowerment***					
If there was a water supply problem in this village/community, how likely is it that people will cooperate to try to solve the problem?	CA8	0.308 *	0.306 *	0.408 *	0.371 *
I have the capacity to achieve my future aims.	CE17	0.576 *	0.588 *	0.457 *	0.430 *
I have the ability to contribute to this community's development.	CE18	0.374 *	0.378 *	0.653 *	0.624 *
People in this community have the capacity to make positive changes by coming together.	CE19	0.584 *	0.579 *	0.670 *	0.638 *
This community needs assistance from others outside the community in order to make positive changes.	CE20	−0.280 *	-	−0.438 *	−0.417 *
People in this community should work together to develop the community.	CE21	0.588 *	0.578 *	0.650 *	0.644 *
We, as a community, can overcome obstacles that we encounter when working toward a common goal.	CE29	0.800 *	0.770 *	0.665 *	0.644 *
People in this community are motivated to achieve common development goals, even when those goals seem challenging.	CE30	0.566 *	0.554 *	0.595 *	0.580 *

Estimation method: WLSMV with sandwich estimator to adjust for non-independence of observations within 30 village clusters. *Matrix:* polychoric correlations. * Statistically significant factor loadings at $p \leq 0.05$.

File S1. Focus group discussion question guide

Introduction

Thank you for agreeing to speak with us today. My name is [Research Assistant/Translator name] and this is my colleague, Allison. We work with WaterAid Cambodia. We are conducting research to learn more about what life is like in your village. We are talking to villages that have participated in the Cambodia Rural Sanitation and Hygiene Improvement Program (CRSHIP). We feel that it is important to speak directly with the people in these villages so that we can learn about their thoughts and experiences. The findings from this study will be used to make recommendations to improve CRSHIP programming.

The discussion will last for about two hours. Please feel free to get up and move around as needed. The restrooms are located [indicate restroom location]. If you need to leave, the exits are located [indicate exit locations].

We want to learn about your thoughts and experiences. There are no right or wrong answers and anything you share will be helpful to our study. I will ask some guiding questions, but please feel free to bring up any additional topics that you find relevant to the discussion. This will be an open discussion, meaning that we will not go around the room to ask each participant each question. Instead, you should feel welcome to join in and provide your own thoughts and experiences when you can. However, everyone's contributions and comments are important to us so it is important that only one person speaks at a time. Additionally, we want to hear as many different perspectives as possible so please feel free to disagree with someone's point of view so long as you are respectful of that individual and their experiences in doing so.

I would like to take notes and record our conversation. The rest of the research team will have access to the notes and recording. However, the notes and recording will not be shared outside of the research team and everything you tell me today will remain confidential. Your name and [village/commune] will be removed from the documents along with any and all other identifying information. We will delete the recording at the end of the study. We also ask that participants do not share what is said here today with anyone outside of our focus group discussion.

I would like to remind everyone that this focus group discussion is completely voluntary. Although we request that you stay for the duration of the discussion, you are free to excuse yourself at any point if you become uncomfortable or need to leave. You are not required to answer any questions that you do not wish to answer or to discuss any topics that you do not wish to discuss. Please let me know if you want to stop recording the discussion at any time. We can also pause the recording at any time if you want to share something that you do not want recorded.

Do you consent to participate in the focus group discussion?

[Obtain verbal confirmation from each participant.]

Is it okay to record the discussion?

If yes: Thank you. I will begin recording now.

If no: That's no problem. I will take notes only.

Does anyone have any questions before we start?

If yes: [address each question]

If no: [proceed to administration of demographic survey]

Demographic Survey

The following survey will be administered before beginning the focus group discussion. The survey is intended to provide basic demographic information of the participants; personally identifiable information will not be collected.

Table 1. Demographic Survey

Age: _______ years	Gender: ☐Male ☐Female
Number of residents in your household (including yourself): ________ people	
Number of years as a resident of this [village/commune]: ________ years	
Household Poverty Level (select one)<br>☐ ID Poor 1 ☐ ID Poor 2 ☐ Not ID Poor	
Education (select one)<br>☐ None, illiterate ☐ Some lower secondary ☐ Some college/university<br>☐ None, literate ☐ Completed lower secondary ☐ Completed college/university<br>☐Some primary ☐ Some upper secondary<br>☐Completed primary ☐ Completed upper secondary	
Latrine access and use<br>1. Do you currently have a latrine that has walls and a squatting surface that provides adequate support for use? (select one)<br>☐Yes, I currently have a latrine<br>☐No, but I did at some point in the past 2 years<br>☐No, but I did at some point more than 2 years ago<br>☐No, I never had a latrine<br>2. Do you share a latrine with other households?<br>☐Yes ☐No<br>3. Do you exclusively defecate in a latrine (i.e. when you are at home as well as when you are at the market, the pagoda, etc.)?<br>☐Yes ☐No<br>4. If no to question 2, where do you defecate?<br>☐ Within the household area ☐ Outside of the household area	

Questions

Warm-up Questions

I'd like to start by giving everyone an opportunity to introduce themselves to the group. Let's go around the room; please share something positive that happened during your week so far. You do not need to say your name.

Next, I will ask an opening question. For this question, I would like everyone to have the opportunity to say something. One person can begin answering the question. When he/she has finished, the person to his/her left can add something new or different. We will continue this way until everyone has had an opportunity to say something.

1. Describe what it is like to live in your village.
    a. What are some of the positive aspects of living in your village?
    b. What are some of the negative aspects of living in your village?

Reflection on CLTS

Next, I'd like to talk about the sanitation and hygiene program implemented by [implementing partner organization name] in this village on [rough triggering date]. This program is called CRSHIP

or CLTS. I'd like to take some time to talk about what it was like in your village before the program. Then we will talk about the program itself. Finally, we can talk about what your village is like now, after the program.

1. Tell me about what this village was like before the sanitation and hygiene program.
    a. What was the sanitation situation like in the village?
        i. Did many households have latrines? Why/why not?
    b. How did villagers feel about sanitation before the program?
    c. How did villagers feel about hygiene before the program?
    d. What was daily life like before the program?
        i. What kinds of interactions did people have with each other?
        ii. How did village members address communal problems?
    e. Did the village have exposure to any other community-based programs before this time? Please explain.

2. What sanitation and hygiene program activities took place in your village during this time?

    [Allow participant to discuss the activities they recall on their own. Tick boxes of the activities that participants mention.]

    a. Who in the village took part in program activities?
        i. Were some people more involved than others? Why/Why not?

    ☐ Village Mapping
    ☐ Calculation of Amount of Feces
    ☐ Transect Walk
    ☐ Analysis of 'Ways of Infection' and How to Prevent Them
    ☐ Calculation of Health Expenses_
    ☐ Village Planning to Achieve ODF Status

3. I'm going to read some sanitation and hygiene activities that you haven't yet mentioned. After each activity I read, please let me know if you remember this activity taking place in your village during the CLTS/CRSHIP.

    [Read only the activities below that were not ticked in question 2. Tick boxes of the activities that participants now remember engaging in.]

☐ <u>Village Mapping:</u>

Facilitator asks villagers to create a large map of the village. Villagers use objects such as stones to identify public places, such as schools, health centers, and water sources. Villagers use more distinct objects to identify their houses on the map.

The facilitator asks how many people use a latrine. For those who do not use a latrine, the facilitator asks that they identify, on the map, where they defecate using objects such as rice husks or sawdust.

☐ <u>Calculation of Amount of Feces:</u>

First, the facilitator and villagers will determine the average weight of feces. The villagers and facilitator will then calculate the amount of feces one person will produce per day, per week, per month, and per year. Finally, the villagers and facilitator will determine the final amount of feces by calculating the number of members in a family and the number of people in the village.

☐ Transect Walk:
In this activity, the facilitator asks villagers to walk around the village and point out where the regular places for open defecation are. The facilitator may also bring a bottle of clean water and see if any participants are willing to drink the water after he/she has stirred the water with feces.

☐ Analysis of 'Ways of Infection' and How to Prevent Them:
The facilitator asks villagers to draw pictures or write the names of infections that they know about. The facilitator will then lead a discussion about how these infections are transmitted and how infectious material can get from places of open defecation back to humans. Finally, the villagers think of ways they can prevent these infections from occurring.

☐ Calculation of Health Expenses
In this activity, the facilitator will ask villagers to talk about expenses incurred by households when a family member becomes ill because of lack of sanitation.

☐ Village Planning to Achieve ODF Status
Finally, the facilitator will ask the villagers to construct a plan to end open defecation in their village entirely. The villagers identify deadlines for when they will become ODF and activities for sanitation behavior change.

Are there any other activities or aspects you remember from this sanitation and hygiene program? Please explain.

4. How did the village react to this sanitation and hygiene program?
    a. Did many people participate? Why/Why not?
    b. What kinds of things did people in the village say about the program?

5. Do you know of any villages that reacted [positively/negatively; the opposite of answer to question 4] to this program?
    a. What makes those villages different from your own village?

6. What changed in the village after this time?
    a. How did sanitation change?
        i. Did people start feeling differently about sanitation after the program? Please explain.
    b. How did hygiene change?

i. Did people start feeling differently about hygiene after the program? Please explain.

c. How did daily life change?
   i. How did interactions among villagers change?
   ii. How did the spirit/atmosphere of the village change?
   iii. Did people begin to work together more or less than they did before the program? Please explain.

7. Are there any activities or meetings that the village conducts now that it did not conduct before?
   a. Tell me about these activities.
   b. Tell me about these meetings.

Perceptions of Collective Efficacy

Next, I'd like to talk about what your village is like now, what it's like to be a member of your village, the different relationships people have, and how village members work together.

1. Do you believe people in the village have the ability to come together and solve a communal problem? Why/Why Not?
   a. What characteristics does your village have that would make you answer in this way?
      i. Does this village have external support from NGOs or the government when facing communal problems or addressing communal goals? Please explain.
         - How does the village react to this support?
         - Do you feel that the village requires this kind of support in order to solve communal problems or address communal goals? Why/why not?

2. Do you know of any villages that have come together to address a communal problem and have [succeeded/failed in doing so; opposite of answer to question 1]?
   a. What about that village is different from yours?
   b. Why do you think the village [was/was not] able to solve the problem?

3. How might you or other people in your village be able to contribute to solving a village problem?
   a. Might you or other people in your village be able to contribute knowledge or information? What kind of knowledge or information?
   b. Might you or other people in your village be able to contribute your skills? What kind of skills?
   c. Might you or other people in your village be able to contribute connections with other people or organizations? What kind of connections?
   d. Are people in this village generally happy to contribute their [knowledge, skills, networks/connections] when there is a problem? Why/why not?

4. Does everyone in the village have the same benefits and opportunities? Why?/Why not?
    a. What kinds of benefits and opportunities do some people in your village have that others do not?
    b. What kinds of benefits and opportunities does almost everyone in your village have in common?
    c. Does everyone in the village receive the same assistance when there is an emergency, such as a drought or a flood? Why?/Why not?

5. Can you tell me about a time a crime was committed in the village?
    a. How did people in the village respond?
    b. How was the issue resolved?

6. How do villagers respond when other households/families in the village have [success/appropriate example of success in this village]?

7. Do people in the village trust village leadership? Why/Why not?
    a. Are village leaders responsive to the needs of village members? Please explain.
    b. Do people in the village trust leaders of community groups or organizations? Why/why not?
    c. Do people in the village trust NGOs that work here? Why/why not?

8. If there were a community development project going on in your village, would you be expected to volunteer your time, labor, or money to contribute to the project? Please explain.
    a. Do the same expectations apply to everyone in the village? Why/Why not?
    b. How would people in the village react if they were asked to contribute their time, labor, or money to contribute to a village development project?
    c. What would happen if someone did not contribute their time, labor, or money to a village development project?
        i. [If 8c involves sanctions/punishments of some kind, ask the question below] Who decides what happens to someone who does not contribute?

Perceptions of Latrine Ownership and Use

Now, I'd like to talk about latrine ownership and latrine use in your village. Think of this village specifically and what tendencies villagers have when it comes to sanitation and hygiene.

1. Where do people in the village normally defecate?
    a. How do people in the village feel about using a latrine to defecate?
        i. Why do you think people feel this way?
    b. How do people in the village feel about openly defecating?
        i. Why do you think people feel this way?

2. What motivates people in your village to have a latrine?

a. What are the challenges people face in buying or building a latrine?

3. What motivates people in your village to maintain and repair their latrines?
    a. What are the challenges people face in maintaining or repairing their latrines?

4. Does your village have rules regarding sanitation and hygiene practices? Please explain.
    a. What about informal, or unspoken, rules?
    b. What would happen if someone in the village were to go against the sanitation and hygiene rules?
        i. Who decides what should happen to someone who goes against the rules?
    c. Who monitors sanitation and hygiene practices?

Concluding Activity

Now, I would like us to engage in a brief activity. During this activity, I will read statements aloud. After each statement, please think silently about whether you agree, disagree, or feel neutral about the statement. Once you have decided, you can stand up and walk to the area of the room that best aligns with the way you feel.

We have placed "Agree," "Disagree," and "Neutral" signs around the room. Think of this as a spectrum. If you strongly agree, stand by the "Agree" sign. If you somewhat agree, stand between the "Agree" and "Neutral" signs. If you are totally neutral, stand by the "Neutral" sign. If you strongly disagree, stand by the "Disagree" sign. If you somewhat disagree, stand between the "Disagree" and "Neutral" signs.

I will read five statements. After each statement, I will pause to allow you to think about whether you agree, disagree, or are neutral, and to move around the room. After each statement, we will also have a brief discussion about why you agree, disagree, or are neutral.

- People in this village share the same goals.
- People in this village can be trusted.
- People in this village generally feel attached to the village and connected to other village members.
- If my family or me were having a hard time with something, such as health or finances, I could count on other people in the village to help us.
- People in this village prioritize their own family's welfare over community development.
- We, as a village, do a good job of organizing ourselves in order to achieve village goals.
- We, as a village, can overcome obstacles that face us when we are working together to solve a problem.

Conclusion

Is there anything else you would like to share today?

If yes: [allow each comment to be shared]

If no: [proceed to conclusion below]

That will conclude our discussion for today. Thank you for participating and for sharing your personal thoughts, feelings, beliefs, and experiences. I would like to remind you that all of the

information shared here today is confidential. Only the research team will have access to the information shared and your names have not been recorded today.

Does anyone have any additional questions?

If yes: [address each question]

If no: Thank you again for participating.

File S2: Key informant interview question guide

Introduction

Thank you for agreeing to speak with us today. My name is [Research Assistant/Translator name] and this is my colleague, Allison. We work with WaterAid Cambodia. We are conducting

research to learn more about what life is like in your village. We are talking to villages that have participated in the Cambodia Rural Sanitation and Hygiene Improvement Program (CRSHIP). We feel that it is important to speak directly with the people in these villages so that we can learn about their thoughts and experiences. The findings from this study will be used to make recommendations to improve CRSHIP programming.

The discussion will last for about one hour. We want to hear about your thoughts and experiences. There are no right or wrong answers and anything you share will be helpful to our study. I will ask some guiding questions, but please feel free to bring up any additional topics that you find relevant to the discussion.

I would like to take notes and record our conversation. The rest of the research team will have access to the notes and recording. However, the notes and recording will not be shared outside of the research team and everything you tell me today will remain confidential. Your name and [village/commune] will be removed from the documents along with any and all other identifying information. We will delete the recording at the end of the study.

I would like to remind you that this interview is completely voluntary. You are not required to answer any questions that you do not wish to answer or to discuss any topics that you do not wish to discuss. Please let me know if you want to stop recording the discussion at any time. We can also pause the recording at any time if you want to share something that you do not want recorded.

Do you consent to participate in the interview?

[Obtain verbal confirmation]

Is it okay to record the discussion?

If yes: Thank you. I will begin recording now.

If no: That's no problem. I will take notes only.

Do you have any questions before we start?

If yes: [address each question]

If no: [proceed to administration of demographic survey]

Demographic Survey

The following survey will be administered before beginning the key informant interview. The survey is intended to provide basic demographic information of the participant; personally identifiable information will not be collected.

Table 1. Demographic Survey

Age: _______ years	Gender: ☐Male ☐Female
Number of residents in your household (including yourself): ________ people	
Number of years as a resident of this [village/commune]: ________ years	
Position held in the [village/commune] (check all that apply):<br>☐ Village chief ☐Commune councilor ☐Other: _____________<br>☐ Village focal person ☐Commune focal person ☐None	
Number of years in this position within this [village/commune]: ________ years	
Household Poverty Level (select one)<br>☐ ID Poor 1 ☐ ID Poor 2 ☐ Not ID Poor	
Education (select one)<br>☐ None, illiterate ☐ Some lower secondary ☐ Some college/university<br>☐ None, literate ☐ Completed lower secondary ☐ Completed college/university<br>☐Some primary ☐ Some upper secondary<br>☐Completed primary ☐ Completed upper secondary	
Latrine access and use<br>1. Do you currently have a latrine that has walls and a squatting surface that provides adequate support for use? (select one)<br>☐Yes, I currently have a latrine<br>☐No, but I did at some point in the past 2 years<br>☐No, but I did at some point more than 2 years ago<br>☐No, I never had a latrine<br>2. Do you share a latrine with other households?<br>☐Yes ☐No<br>3. Do you exclusively defecate in a latrine (i.e. when you are at home as well as when you are at the market, the pagoda, etc.)?<br>☐Yes ☐No<br>4. If no to question 2, where do you defecate?<br>☐ Within the household area ☐ Outside of the household area	

Questions

Warm-up Questions

I'd like to start by learning a bit more about you and your work.

2. Tell me about your role as [position held]?
    a. How does this role connect to the leadership and/or governance of [village name]?
    b. Can you tell me about what you do during a typical week as [position held]?

Reflection on CLTS

Next, I'd like to talk about the sanitation and hygiene program implemented by [implementing partner organization name] in this village on [rough triggering date]. This program is called CRSHIP or CLTS. I'd like to take some time to talk about what it was like in your village before the program.

Then we will talk about the program itself. Finally, we can talk about what your village is like now, after the program.

8. Tell me about what this village was like before the sanitation and hygiene program?
    a. What was the sanitation situation like in the village?
        i. Did many households have latrines? Why/why not?
    b. How did villagers feel about sanitation before the program?
    c. How did villagers feel about hygiene before the program?
    d. What was daily life like before the program?
        i. What kinds of interactions did people have with each other?
        ii. How did village members address communal problems?
    e. Did the village have exposure to any other community-based programs before this time? Please explain.

9. What sanitation and hygiene program activities took place in your village during this time?
    [Allow participant to discuss the activities they recall on their own. Tick boxes of the activities that participants mention.]

    ☐ Village Mapping
    ☐ Calculation of Amount of Feces
    ☐ Transect Walk
    ☐ Analysis of 'Ways of Infection' and How to Prevent Them
    ☐ Calculation of Health Expenses_
    ☐ Village Planning to Achieve ODF Status

10. I'm going to read some sanitation and hygiene activities that you haven't yet mentioned. After each activity, I read, please let me know if you remember this activity taking place in your village during the CLTS/CRSHIP.
    [Read only the activities below that were not ticked in question 2. Tick boxes of the activities that participants now remember engaging in.]

☐ Village Mapping:

Facilitator asks villagers to create a large map of the village. Villagers use objects such as stones to identify public places, such as schools, health centers, and water sources. Villagers use more distinct objects to identify their houses on the map.

The facilitator asks how many people use a latrine. For those who do not use a latrine, the facilitator asks that they identify, on the map, where they defecate using objects such as rice husks or sawdust.

☐ Calculation of Amount of Feces:

First, the facilitator and villagers will determine the average weight of feces. The villagers and facilitator will then calculate the amount of feces one person will produce per day, per week, per month, and per year. Finally, the villagers and

facilitator will determine the final amount of feces by calculating the number of members in a family and the number of people in the village.

☐ Transect Walk:

In this activity, the facilitator asks villagers to walk around the village and point out where the regular places for open defecation are. The facilitator may also bring a bottle of clean water and see if any participants are willing to drink the water after he/she has stirred the water with feces.

☐ Analysis of 'Ways of Infection' and How to Prevent Them:

The facilitator asks villagers to draw pictures or write the names of infections that they know about. The facilitator will then lead a discussion about how these infections are transmitted and how infectious material can get from places of open defecation back to humans. Finally, the villagers think of ways they can prevent these infections from occurring.

☐ Calculation of Health Expenses

In this activity, the facilitator will ask villagers to talk about expenses incurred by households when a family member becomes ill because of lack of sanitation.

☐ Village Planning to Achieve ODF Status

Finally, the facilitator will ask the villagers to construct a plan to end open defecation in their village entirely. The villagers identify deadlines for when they will become ODF and activities for sanitation behavior change.

Are there any other activities or aspects you remember from this sanitation and hygiene program? Please explain.

11. How did the village react to this sanitation and hygiene program?
    a. Did many people participate? Why/Why not?
    b. What kinds of things did people in the village say about the program?

12. Do you know of any villages that reacted [positively/negatively; the opposite of answer to question 4] to this program?
    a. What makes those villages different from your own village?

13. What changed in the village after this time?
    a. How did sanitation change?
        i. Did people start feeling differently about sanitation after the program? Please explain.
    b. How did hygiene change?
        i. Did people start feeling differently about hygiene after the program? Please explain.
    c. How did daily life change?

i. How did interactions among villagers change?
ii. How did the spirit/atmosphere of the village change?
iii. Did people begin to work together more or less than they did before the program? Please explain.

14. Are there any activities or meetings that the village conducts now that it did not conduct before?
    a. Tell me about these activities.
    b. Tell me about these meetings.

Village-level Social Norms

Now, I would like us to talk about the beliefs of people in the village around sanitation and hygiene. Think of this village specifically and what tendencies villagers have when it comes to sanitation and hygiene.

1. Where do people in the village normally defecate?
    a. How do people in the village feel about using a latrine to defecate?
        i. Why do you think people feel this way?
    b. How do people in the village feel about openly defecating?
        i. Why do you think people feel this way?

2. What motivates people in your village to have a latrine?
    a. What are the challenges people face in buying or building a latrine?

3. What motivates people in your village to maintain and repair their latrines?
    a. What are the challenges people face in maintaining or repairing their latrines?

4. Does your village have rules regarding sanitation and hygiene practices? Please explain.
    a. What about informal, or unspoken, rules?
    b. What would happen if someone in the village were to go against the sanitation and hygiene rules?
        i. [Ask this question only if the participant answers question 3b by saying that something negative would happen.]
        Who decides what should happen to someone who goes against the rules?
    c. Who monitors sanitation and hygiene practices?

Perceptions of Collective Efficacy

Next, I'd like to talk about what your village is like now, what it's like to be a member of your village, the different relationships people have, and how village members work together.

9. Do you believe people in the village have the ability to come together and solve a communal problem? Why/Why Not?

  b. Do you know of any villages that have come together to address a communal problem and have [succeeded/failed in doing so; opposite of answer to question 1]?
     i. What about that village is different from yours?
     ii. Why do you think the village [was/was not] able to solve the problem?

10. How might you be able to contribute to solving a village problem?
  e. Might you be able to contribute knowledge or information? What kind of knowledge or information?
  f. Might you be able to contribute your skills? What kind of skills?
  g. Might you be able to contribute connections with other people or organizations? What kind of connections?

11. How similar are the various households/families in the village?
  a. In what ways are they similar?
  b. In what ways are they different?
  c. What kinds of things do people usually agree about?
  d. What kinds of things do people usually disagree about?

12. Does everyone in the village have the same benefits and opportunities? Why?/Why not?
  a. What kinds of benefits and opportunities do some people have that others do not?
  b. What kinds of benefits and opportunities does almost everyone have in common?
  c. Does everyone in the village receive the same assistance when there is an emergency, such as a drought or a flood? Why?/Why not?

13. Can you tell me about a time a crime was committed in the village?
  a. How did people in the village respond?
  b. How was the issue resolved?

14. How do villagers respond when other households/families in the village have [success/appropriate example of success in this village]?

15. If there were a community development project going on in your village, would you be expected to volunteer your time, labor, or money to contribute to the project? Please explain.
  a. Do the same expectations apply to everyone in the village? Why/Why not?
  b. How would you react if they were asked to contribute their time, labor, or money to contribute to a village development project?
  c. What would happen if someone did not contribute their time, labor, or money to a village development project?
     i. [If 7c involves sanctions/punishments of some kind, ask the question below]
        Who decides what happens to someone who does not contribute?

External Influences/Systems

Next, I would like to talk to you about external factors that may influence what life is like in your village, how people work together, and how people make decisions about sanitation and hygiene. We will talk about how the government, the economy, NGOs, and history might impact daily life in the village.

1. Do you feel that villagers trust the people that come into the village to implement sanitation and hygiene programs? Why/Why not?

2. Do you feel that villagers trust government officials who promote sanitation and hygiene in the provinces? Why/Why not?

3. How do government policies affect sanitation and hygiene in the village?
    a. In what ways do government policies facilitate good sanitation and hygiene in the village?
    b. In what ways do government policies act as barriers to good sanitation and hygiene in the village?
    c. How do government policies affect the ability of village members to work together?
        i. Are community groups affected by government policies? Why/why not?

4. Tell me about the economy of the village.
    a. In what ways does the economy facilitate good sanitation and hygiene in the village?
    b. In what ways does the economy act as a barrier to good sanitation and hygiene in the village?
        i. How might the village be able to overcome these barriers?
    c. How does the economy of the village affect decision-making with regards to sanitation and hygiene?
        i. Does the economy of the village affect the ability of village members to work together? Please explain.

5. How do NGOs in the region affect the sanitation and hygiene situation in the village?
    a. In what ways do NGOs facilitate good sanitation and hygiene in the village?
    b. In what ways do NGOs act as a barrier to good sanitation and hygiene in the village?
        i. How might the village be able to overcome these barriers?
    c. How do NGOs affect decision-making with regards to sanitation and hygiene?
    d. How do NGOs affect the village's ability to solve communal problems?
6. Tell me about some of the history of this village.
    a. How does this history impact the village today?
        i. In what ways is the village positively affected by its history?
        ii. In what ways is the village negatively affected by its history?

Conclusion

Is there anything else you would like to share today?

If yes: [allow each comment to be shared]

If no: [proceed to conclusion below]

That will conclude our discussion for today. Thank you for participating and for sharing your personal thoughts and experiences. I would like to remind you that all of the information shared here today is confidential. Only the research team will have access to the information shared and your names have not been recorded today.

Does you have any additional questions?

If yes: [address each question]

If no: Thank you again for participating.

© 2020 by the authors. Licensee MDPI, Basel, Switzerland. This article is an open access article distributed under the terms and conditions of the Creative Commons Attribution (CC BY) license (http://creativecommons.org/licenses/by/4.0/).
